# Consecutive Hypoxia Decreases Expression of *NOTCH3*, *HEY1*, *CC10*, and *FOXJ1* via *NKX2-1* Downregulation and Intermittent Hypoxia-Reoxygenation Increases Expression of *BMP4*, *NOTCH1*, *MKI67*, *OCT4*, and *MUC5AC* via *HIF1A* Upregulation in Human Bronchial Epithelial Cells

**DOI:** 10.3389/fcell.2020.572276

**Published:** 2020-09-04

**Authors:** Yung-Yu Yang, Chao-Ju Lin, Cheng-Chin Wang, Chieh-Min Chen, Wen-Jen Kao, Yi-Hui Chen

**Affiliations:** ^1^Department of General Medicine, Tri-Service General Hospital, National Defense Medical Center, Taipei, Taiwan; ^2^Graduate Institute of Aerospace and Undersea Medicine, National Defense Medical Center, Taipei, Taiwan; ^3^Section of Respiratory Therapy, Rueifang Miner Hospital, New Taipei City, Taiwan; ^4^Graduate Institute of Microbiology and Immunology, National Defense Medical Center, Taipei, Taiwan

**Keywords:** air-liquid interface, apoptosis, differentiation, *FOXJ1*, human bronchial epithelial cells, *MUC5AC*, proliferation, stem/progenitor cell marker

## Abstract

Previous studies have shown that the experimental models of hypoxia-reoxygenation (H/R) mimics the physiological conditions of ischemia-reperfusion and induce oxidative stress and injury in various types of organs, tissues, and cells, both *in vivo* and *in vitro*, including human lung adenocarcinoma epithelial cells. Nonetheless, it had not been reported whether H/R affected proliferation, apoptosis, and expression of stem/progenitor cell markers in the bronchial epithelial cells. In this study, we investigated differential effects of consecutive hypoxia and intermittent 24/24-h cycles of H/R on human bronchial epithelial (HBE) cells derived from the same-race and age-matched healthy subjects (i.e., NHBE) and subjects with chronic obstructive pulmonary disease (COPD) (i.e., DHBE). To analyze gene/protein expression during differentiation, both the NHBE and DHBE cells at the 2nd passage were cultured at the air-liquid interface (ALI) in the differentiation medium under normoxia for 3 days, followed by either culturing under hypoxia (1% O_2_) for consecutively 9 days and then returning to normoxia for another 9 days, or culturing under 24/24-h cycles of H/R (i.e., 24 h of 1% O_2_ followed by 24 h of 21% O_2_, repetitively) for 18 days in total, so that all differentiating HBE cells were exposed to hypoxia for a total of 9 days. In both the normal and diseased HBE cells, intermittent H/R significantly increased *HIF1A*, *BMP4*, *NOTCH1*, *MKI67*, *OCT4*, and *MUC5AC* expression, while consecutive hypoxia significantly decreased *NKX2-1*, *NOTCH3*, *HEY1*, *CC10*, and *FOXJ1* expression. Inhibition of *HIF1A* or *NKX2-1* expression by siRNA transfection respectively decreased *BMP4*/*NOTCH1*/*MKI67*/*OCT4*/*MUC5AC* and *NOTCH3*/*HEY1/CC10/FOXJ1* expression in the HBE cells cultured under intermittent H/R to the same levels under normoxia. Overexpression of *NKX2-1* via cDNA transfection caused more than 2.8-fold increases in *NOTCH3*, *HEY1*, and *FOXJ1* mRNA levels in the HBE cells cultured under consecutive hypoxia compared to the levels under normoxia. Taken together, our results show for the first time that consecutive hypoxia decreased expression of the co-regulated gene module *NOTCH3/HEY1/CC10* and the ciliogenesis-inducing transcription factor gene *FOXJ1* via *NKX2-1* mRNA downregulation, while intermittent H/R increased expression of the co-regulated gene module *BMP4/NOTCH1/MKI67/OCT4* and the predominant airway mucin gene *MUC5AC* via *HIF1A* mRNA upregulation.

## Introduction

Previous studies have demonstrated that the intracellular effects and cellular injury caused by hypoxia-reoxygenation (H/R) *in vitro* are similar to the responses induced by ischemia-reperfusion *in vivo* (Samaja and Milano, 2015; Gerõ, 2017). Under hypoxia, cells undergo energy depletion and transform from aerobic mitochondrial respiration into anaerobic metabolism, which induces intracellular acidosis via increasing intracellular concentrations of lactate and protons, and results in disequilibrium of the cytoplasmic concentrations of sodium and calcium ions (Gourdin and Dubois, 2017). Upon reperfusion or reoxygenation after a period of hypoxia, there will be intramitochondrial calcium overload and accumulation of reactive oxygen species (ROS) due to a sudden increase of intramitochondrial oxygen molecules and dramatic decrease of intracellular protons, and hence leading to opening of the mitochondrial permeability transition pore, which then induces cell apoptosis (Kalogeris et al., 2012; Gerõ, 2017). It has been reported that ischemia-reperfusion is a common hemorheological situation associated with solid organ transplantation, including lung transplantation (Fiser et al., 2002; de Perrot et al., 2003; Cicco et al., 2005; Kosieradzki and Rowinski, 2008; Lobo et al., 2014; Chen and Date, 2015; Laubach and Sharma, 2016), and that interruption of bronchial artery circulation during lung transplantation is highly associated with airway ischemia/hypoxia, pulmonary fibrosis, bronchiolitis obliterans syndrome, susceptibility to chronic rejection, and low long-term survival (Nicolls and Zamora, 2010; Wilkes, 2010; Tong et al., 2015).

Previous *in vivo* studies of short-term intermittent hypoxia-reoxygenation ranging from 10 min to 5 days using the rat or mouse models have reported significantly increased levels of oxidative stress and expression of inflammatory and pro-apoptotic genes (Shen et al., 2008; Rus et al., 2010; Wollen et al., 2013; Gonchar and Mankovska, 2017; Rognlien et al., 2017). In addition, *in vitro* studies of H/R using the adenocarcinomic human lung epithelial cell lines A549 and H441 also showed increased oxidative stress and expression of nuclear NF-κB and the pro-inflammatory cytokines IL-1β and IL-6 as well as decreased oxygen consumption and MnSOD activity and expression of the anti-inflammatory cytokine IL-10 and the alveolar surfactant proteins SP-A, SP-D, SP-B, and SP-C (Li et al., 2002; Liu et al., 2015). Nonetheless, H/R did not significantly influence the viability of both A549 and H441 cells in comparison with the air-exposed controls (Li et al., 2002; Liu et al., 2015). Therefore, exposure to intermittent H/R has been shown to increase levels of oxidative stress, inflammation and apoptosis in the airway epithelial cells both *in vivo* and *in vitro*.

In addition to H/R, consecutive hypoxia for a long period has also been reported to modulate the inflammatory functions and oxidant-induced cell injury in airway epithelial cells (Olson et al., 2011; Pahlman et al., 2015; Pandya et al., 2016; Polke et al., 2017). It was shown that consecutive hypoxia for longer than 24 h contributed to the pathogenesis of chronic pulmonary diseases via both the HIF1α-dependent downregulation of the complement regulatory protein CD55 and several inflammatory cytokines, including IL-6, IP-6, IP-10, and hBD-2, as well as the TGFβ1-dependent downregulation of the secretory leukocyte protease inhibitor, in human airway epithelial cells (Pahlman et al., 2015; Pandya et al., 2016; Polke et al., 2017). Continuous exposure to hypoxia for 24 h was also reported to inhibit active sodium absorption and the HIF2α- and NF-κB-dependent bacterial infection (Tomlinson et al., 1999; Schaible et al., 2013), while hypoxia for 12 h was shown to increase HIF1α-dependent miR-200b expression and decrease expression of the cystic fibrosis transmembrane conductance regulator (CFTR) (Bartoszewska et al., 2017), and hypoxia for 6 h was sufficient to significantly ameliorate the oxidant-induced epithelial barrier dysfunction (Olson et al., 2011).

Long-term hypoxia has also been shown to regulate mucus secretion and arrangements of goblet cells as well as proliferation and differentiation of airway epithelial cells (Konrádová et al., 2002; Polosukhin et al., 2011; Leahy, 2012; Gerovac et al., 2014; Torres-Capelli et al., 2016). *In vivo*, consecutive exposure to 10% O_2_ was reported to significantly increase proliferation of bronchial epithelial cells in mice, predominantly Clara cells, via the Hif2α−FoxM1−Relmα/β regulatory pathway, after 3 days of exposure (Torres-Capelli et al., 2016), while consecutive exposure to 10% O_2_ for 4 days was shown to significantly increase the percentages of mucus-discharging and mucous gland-forming goblet cells in the tracheal epithelium in rabbits (Konrádová et al., 2002). *In vitro*, consecutive exposure of human bronchial epithelial cells in air-liquid interface (ALI) cultures to 1% O_2_ for 14−28 days or to 0.5% O_2_ for 21 days significantly decreased numbers of FOXJ1^+^ ciliated cells and increased numbers of MUC5AC^+^ goblet cells (Polosukhin et al., 2011; Gerovac et al., 2014), while exposure to 5% O_2_ for 6 − 12 h was sufficient to induce de-differentiation of murine airway epithelial cells in ALI cultures (Leahy, 2012). In addition, in large airway specimens obtained from patients with chronic obstructive pulmonary disease (COPD) but not from smokers without COPD, nuclear HIF1α staining was detected in areas of goblet cell hyperplasia (Polosukhin et al., 2011). According to previous studies, in comparison with the consecutive hypoxia for 24 h, 24/24-h cycles of H/R led to a significantly lower level of the cellular ATP content and a significantly higher level of lactate dehydrogenase, indicating that intermittent H/R significantly caused a higher level of oxidative stress compared to consecutive hypoxia (Li et al., 2002). Given that long-term exposure to consecutive hypoxia (e.g., 14−28 days of ALI cultures under 1% O_2_ or 0.5% O_2_) significantly altered the mucociliary differentiation of bronchial epithelial cells, and that during ALI culturing, the period between day 6 and day 10 was most critical for ciliated cell differentiation (Polosukhin et al., 2011; Leahy, 2012; Gerovac et al., 2014), we were interested in further elucidating whether intermittent H/R exerts even more exacerbated effects compared to consecutive hypoxia on altering the mucociliary differentiation of airway epithelial cells in the ALI cultures.

In this study, we found that the human bronchial epithelial (HBE) cells which were ALI-cultured and consecutively exposed to 1% O_2_ for 18 days failed to differentiate and express the ciliogenesis-inducing transcription factor FOXJ1 (You et al., 2004; Yu et al., 2008) and the major secreted airway mucin MUC5AC (Hovenberg et al., 1996; Lillehoj et al., 2013). Consecutive exposure to 1% O_2_ for 9 days followed by exposure to 21% O_2_ for another 9 days significantly decreased FOXJ1 expression in both the differentiated HBE cells derived from healthy donors (i.e., normal HBE or NHBE cells) and the differentiated HBE cells derived from COPD subjects (i.e., diseased HBE or DHBE cells) but significantly increased MUC5AC expression in the differentiated DHBE instead of NHBE cells. On the other hand, intermittent H/R exposure to 24/24-h cycles of 1 and 21% O_2_ for 18 days in total dramatically increased MUC5AC expression in both the differentiated NHBE and DHBE cells and significantly increased FOXJ1 expression in the differentiated NHBE but not DHBE cells. We further showed that, in the ALI-cultured NHBE and DHBE cells, the changes in the expression levels of MUC5AC mRNA and protein were concordant with the changes in the expression levels of HIF1A, BMP4 and MKI67 mRNAs and proteins, while the changes in the expression levels of FOXJ1 mRNA and protein were concordant with the changes in the expression levels of NKX2-1 and HEY1 mRNAs and proteins. Inhibition of *HIF1A* mRNA expression by transfection with *HIF1A* siRNA was sufficient to suppress expression of *BMP4*, *NOTCH1*, *MKI67*, and *MUC5AC* mRNAs, while inhibition or overexpression of *NKX2-1* mRNA levels via transfection with the *NKX2-1* siRNA or cDNA, respectively, led to significant decreases or increases in the mRNA levels of *NOTCH3*, *HEY1* and *FOXJ1* in the ALI-cultured NHBE and DHBE cells. In addition, in the submerged cultures of HBE cells, the expression levels of the stem/progenitor cell markers OCT4 and CC10 were respectively regulated by the levels of *HIF1A* and *NKX2-1* mRNAs. Taken together, we found for the first time that consecutive hypoxia decreased expression of the co-regulated gene module *NOTCH3/HEY1/CC10* and the ciliogenesis-inducing transcription factor gene *FOXJ1* via *NKX2-1* mRNA downregulation, while intermittent H/R increased expression of the co-regulated gene module *BMP4/NOTCH1/MKI67/OCT4* and the main secreted airway mucin gene *MUC5AC* via *HIF1A* mRNA upregulation.

## Materials and Methods

### The Submerged and Air-Liquid Interface (ALI) Cultures of Human Bronchial Epithelial (HBE) Cells Under Normoxia, Intermittent H/R or Consecutive Hypoxia

The human bronchial/tracheal epithelial (HBE) cells for B-ALI^TM^ culture used in this study were purchased from Lonza Walkersville Inc. in Walkersville, MD, United States, and contained three different normal HBE (i.e., NHBE) primary cell lines derived from three distinct healthy Caucasian donors, including one 66-year-old and two 67-year-old donors, and three different diseased HBE (i.e., DHBE) primary cell lines derived from three distinct COPD patients who were respectively 65-, 66-, and 59-year-old Caucasians. The detailed information about the smoking habit and history of cardiovascular diseases for each HBE cell donor is listed in Table 1. These HBE cells were originally derived from biopsies taken during tracheo-bronchoscopy procedures from the trachea-bronchial junction surrounding the airway bifurcation.

**TABLE 1:** Description of HBE cells and information of cell donors.

**Cell type**	**Product code**	**Lot number**	**Cell doubling time**	**Donor Age**	**Gender**	**Race**	**Smoking history**	**Other disease history**
NHBE1	CC-2540S	0000436081	21 h	66 Y	Male	Caucasian	No	Hypertension
NHBE2	CC-2540S	0000446317	25 h	67 Y	Male	Caucasian	Yes (2 years)	No
NHBE3	CC-2540S	0000497122	29 h	66 Y	Female	Caucasian	No	No
DHBE1	00195275S	0000370751	27 h	65 Y	Female	Caucasian	No	Hypertension
DHBE2	00195275S	0000430905	21 h	66 Y	Male	Caucasian	Yes (48 years)	Hypertension
DHBE3	00195275S	0000436083	22 h	59 Y	Male	Caucasian	Yes (30 yrs)	Heart disease

The purchased HBE cells were all at the first passage (i.e., Passage 1 or P1) and the frozen cell densities were all between 5 × 10^5^ and 1.1 × 10^6^ cells/ml. The P1 HBE cells were thawed and cultured in the Bronchial Epithelial Growth Media (BEGM^TM^ BulletKit^TM^, Lonza) for 8–10 days in the T175 Nunc^TM^ EasYFlask^TM^ cell culture flasks (Thermo Fisher Scientific) at 37°C with 5% CO_2_ till 100% confluence, with the BEGM medium changed every other day. After reaching confluency, each primary HBE cell line was separated into 10–12 biobanking cryotubes (Nunc^TM^, Thermo Fisher Scientific), depending on the total cell number, with each tube containing 1.0 × 10^6^ cells/ml mixed with the Stem-Cellbanker^®^ cryopreservation solution (ZENOAQ, Japan), followed by cryopreservation in liquid nitrogen under −195°C. For subsequent experiments of both submerged and ALI cultures, one cryovial of each NHBE and DHBE cell line was thawed for each experiment. Four cryovials in total for each NHBE and DHBE cell line were independently thawed and cultured for the immunofluorescence staining analyses, and three cryovials in total for each NHBE and DHBE cell line were independently thawed and cultured for the real-time qPCR analyses.

For submerged cultures, the frozen P2 HBE cells in the cryovials were sucultured at a density of 2.5 × 10^4^ cells/ml into 10-cm petri dishes containing 16-ml BEGM medium for 6 days in total, followed by mRNA extraction and qPCR analyses, or subcultured at a density of 3500 cells/cm into 24-well cell culture plates containing 2-ml BEGM medium per well for 6 days in total, followed by immunofluorescence staining and image analyses. The numbers and densities of HBE cells were counted by both the hemocytometer and ADAM-MC automatic cell counter by NanoEnTek Inc. (Pleasanton, CA, United States).

During the 6 days of subculturing, the P3 NHBE and DHBE cells were respectively separated into three groups and placed in two separate tri-gas SMA-165DS incubators (ASTEC Co., Ltd., Japan), with one incubator set up with 5%CO_2_/balance air (normoxia), and the other one set up with 1% O_2_/5% CO_2_/balance N_2_ (hypoxia). The “consecutive hypoxia” groups of NHBE and DHBE cells were placed in the hypoxia incubator consecutively for three days, followed by moving to the normoxia incubator for another three days, while the “intermittent hypoxia-reoxygenation (H/R)” groups of NHBE and DHBE cells were cultured under 24/24-h cycles of hypoxia-reoxygenation, namely, were placed in the hypoxia incubator for 24 h, followed by moving to the normoxia incubator for 24 h, and then moving back to the hypoxia incubator for another 24 h, and so forth, for 6 days in total.

For air-liquid interface (ALI) cultures to induce cell differentiation, after the first subculturing, the P2 HBE cells were incubated at a density of 5 × 10^5^ cells/ml and submerged in 400 μl of the CnT Prime Airway proliferation medium CnT-PR-A (CELLnTEC advanced cell systems AG) within each 12-mm Millicell^®^ hydrophilic PTFE cell culture insert with a pore size of 0.4 μm (Merck Millipore), which was individually placed in each well of the 24-well cell culture plates. After the monolayer of the HBE cells grown on the PTFE membrane reached 100% confluence within 2–3 days, the HBE cell cultures were separated into three groups and placed in two separate tri-gas SMA-165DS incubators (ASTEC Co., Ltd., Japan), with one incubator set up with 5%CO_2_/balance air (normoxia), and the other one set up with 1% O_2_/5% CO_2_/balance N_2_ (hypoxia), and differentiation of the HBE cells was induced by replacing the original CnT-PR-A proliferation medium with the CnT Prime Airway Differentiation medium CnT-PR-AD containing 1 mM CaCl_2_ (CELLnTEC advanced cell systems AG) both inside and outside the Millicell^®^ inserts and incubated for 15–16 h.

In order to initiate ALI-induced differentiation of the HBE cells, all medium from the inside of the Millicell^®^ inserts was aspirated and the medium outside the inserts was replaced with fresh CnT-PR-AD medium containing 1 mM CaCl_2_. The ALI cultures were continued for 18 days in total with the CnT-PR-AD medium changed daily and the surface of the Millicell^®^ inserts kept dry throughout the whole process. The “9-day consecutive hypoxia” group of HBE cells was placed in the hypoxia incubator consecutively for 9 days, followed by moving to the normoxia incubator for another 9 days, while the “18-day consecutive hypoxia” group of HBE cells was placed in the hypoxia incubator consecutively for 18 days and then directly proceeded to immunostaining analyses or total RNA extraction for qPCR analyses without returning to the normoxia incubator. The “intermittent hypoxia-reoxygenation (H/R)” group of HBE cells was cultured under 24/24-h cycles of hypoxia-reoxygenation, namely, was placed in the hypoxia incubator for 24 h, followed by moving to the normoxia incubator for 24 h, and then moving back to the hypoxia incubator for another 24 h, and so forth, for 18 days in total.

### Immunofluorescence Staining and Image Analyses

For immunofluorescence staining, both the undifferentiated HBE cells in submerged cultures in the 24-well plates for 6 days and the differentiated HBE tissues cultured at the ALI in the Millicell^®^ inserts were first washed in 1x PBS at pH 7.4 for 5 min followed by submersion and fixation in 4% paraformaldehyde (PFA) (Sigma-Aldrich) in PBS for 20 min. The fixed HBE cells or tissues were then washed in 1x PBS, permeabilized with 0.5% Triton X-100, followed by blocking with 10% bovine serum albumin (BSA, Sigma-Aldrich) in 1x PBS before immunostained with the following primary antibodies diluted in 1x PBS at 4°C overnight: anti-BMP4 (catalog number PA5-27288; rabbit polyclonal; 1:100 dilution; Thermo Fisher Scientific), anti-CC10 (catalog number sc-9772; goat polyclonal; 1:50 dilution; Santa Cruz Biotechnology), anti-cleaved Caspase-3 (catalog number 9664L; rabbit monoclonal with the clone number 5A1E; 1:400 dilution; Cell Signaling Technology), anti-FOXJ1 (catalog number sc-53139; mouse monoclonal with the clone number 3-19; 1:100 dilution; Santa Cruz Biotechnology), anti-HEY1 (catalog number ab22614; rabbit polyclonal; 1:200 dilution; Abcam), anti-HIF1α (catalog number sc-53546; mouse monoclonal with the clone number H1alpha 67; 1:100 dilution; Santa Cruz Biotechnology), anti-Ki67 (catalog number AF7649; sheep polyclonal; 1:20 dilution; R&D Systems, Inc.), anti-MUC5AC (catalog number sc-16910; goat polyclonal; 1:100 dilution; Santa Cruz Biotechnology), anti-NKX2-1 (catalog number sc-53136; mouse monoclonal with the clone number 8G7G3/1; 1:100 dilution; Santa Cruz Biotechnology), anti-OCT4 (catalog number ab27985; goat polyclonal; 1:100 dilution; Abcam). For isotype negative controls, goat polyclonal IgG (catalog number ab37373), mouse monoclonal IgG1 (catalog number ab18447; with the clone number MG1-45), and rabbit polyclonal IgG (catalog number ab171870) were all obtained from Abcam and applied at the same concentration as the respective primary antibodies.

On the second day, the cells were also washed in 1x PBS and blocked with 10% BSA in 1x PBS before incubation at room temperature for 1 h with the following IgG (H + L) cross-adsorbed secondary antibodies conjugated with fluorophores obtained from Thermo Fisher Scientific: donkey anti-goat Alexa Fluor^®^ 555, donkey anti-goat Alexa Fluor^®^ 647, donkey anti-mouse Alexa Fluor^®^ 488, donkey anti-rabbit Alexa Fluor^®^ 555, donkey anti-rabbit Alexa Fluor^®^ 647, and donkey anti-sheep Alexa Fluor^®^ 488. After 1 h, the cells were washed in 1x PBS again, followed by counter-staining with 10 μg/ml of (DAPI; 1:1000 dilution; Thermo Fisher Scientific) in 1x PBS for 15 min.

Subsequently, the cells were washed and stored in 1x PBS, followed by photographing under the Zeiss LSM 880 with Airyscan confocal laser scanning microscope using the parameter setting of 405/488/543/633-nm laser lines and the Z-Stack multidimensional image acquisition function with the Argon, HeNe594 and HeNe633 lasers on. To ensure a consistent setting of the exposure time, the pinhole values were always set at one Airy Unit, and the gain values for all channels scanning the differentiated HBE tissues in the ALI cultures were always set at 2.0, while the gain values for different channels scanning the undifferentiated HBE cells in the submerged cultures were set at 1.5. Both isotype negative controls and no primary antibody controls showed no specific staining with only very few background signals (data not shown). The images of Z-stack slices were obtained by setting up the scanning interval (e.g., the Z-distance between slices) at 5 μm, and hence the entire scanning range was between 40 and 60 μm (e.g., approximately 8–12 slices in total) for the HBE cells in submerged cultures, and was between 80 and 130 μm (e.g., approximately 16–26 slices in total) for the HBE cells in ALI cultures.

The image files of all Z-stack slices obtained above were then input into the Fiji (ImageJ) software (National Institutes of Health, United States) for quantitative analyses and comparison among different groups of HBE cells/tissues, which were respectively cultured under normoxia, intermittent H/R or consecutive hypoxia, and transfected with or without *HIF1A* siRNA (under intermittent H/R) or *NKX2-1* siRNA or *NKX2-1* cDNA (under consecutive hypoxia). Briefly, for cell counting, the numbers of HBE cells or nuclei stained with different colors of fluorescence (green, magenta or red) and the total numbers of DAPI-stained nuclei were respectively counted on the image files of each experimental group. The average percentages of positive HBE cells or nuclei in each experimental group were calculated as: (the total numbers of cells or nuclei stained with green, magenta or red fluorescence)/(the total numbers of DAPI^+^ nuclei) × 100%. For the differentiated HBE tissues grown in the ALI cultures for 21 days in total, the total cell numbers in each Z-stack slice image under the 20X objective lens were respectively 1891 ± 223 and 2684 ± 205 for the NHBE and DHBE tissues cultured under normoxia, and respectively 2664 ± 247 and 3248 ± 312 for the NHBE and DHBE tissues cultured under intermittent H/R, and respectively 2403 ± 251 and 3172 ± 289 for the NHBE and DHBE tissues cultured under 9-day consecutive hypoxia. For comparison of fluorescent intensities, a specified unit of area (e.g., a defined range covering the cytoplasm or nucleus) in the NHBE cells/tissues cultured under normoxia was set up to quantify the fluorescent intensity within the area, which was recorded as a standard value. The same size of the unit was then picked up from the NHBE cells/tissues in the other experimental groups (i.e., cultured under intermittent H/R or consecutive hypoxia) or from the DHBE cells/tissues (cultured under normoxia, intermittent H/R or consecutive hypoxia), followed by quantification of fluorescent intensities within these units of areas and comparison with the standard value. The relative intensity of a specific protein marker within an experimental group was calculated by the summation of all values of the relative immunofluorescence intensities obtained from all units of areas on all image files within that group, and was quantified as a fold-change of the standard value.

### Total RNA Extraction and RNA Quality Assessment

For cell/tissue lysis and total RNA extraction, all of the following procedures were performed on ice: The BEGM^TM^ growth medium or CnT-PR-AD differentiation medium was carefully aspirated from the 10-cm petri dishes or 24-well cell culture plates, respectively, without touching the attached HBE cells/tissues, and then 3 ml of TRIzol^TM^ Reagent was added directly to the bottom of the 10-cm petri dishes, while 1 ml of TRIzol^TM^ Reagent was added to the PTFE membranes of the Millicell^®^ inserts, followed by leaving the petri dishes or 24-well plates on ice for 5 min to lyse the cells, and subsequently pipetting up and down several times to homogenize and using cell scrapers to scrape the HBE cells/tissues attached to the dishes or inserts several times within 5 s (Nunc^TM^ Cell Scrapers from Thermo Fisher Scientific were used for 10-cm petri dishes and Mini Cell Scrapers from Biotium/VWR were used for 24-well cell culture plates).

Each 1 ml of the homogenized TRIzol^TM^ solution containing lysed HBE cells/tissues was then collected into a 1.5-ml Eppendorf^®^ microcentrifuge tube and the total RNA was extracted according to the manufacturer’s instructions. To determine the RNA yield and purity, the Thermo Scientific^TM^ NanoDrop^TM^ One UV-Visible spectrophotometer system (Thermo Fisher Scientific) was applied according to the manufacturer’s instructions. Concentrations of nucleic acids can be calculated using the reading of ultraviolet (UV) absorbance at 260 nm and a conversion factor based on the extinction coefficient for each nucleic acid. In our study, the total amount of each extracted RNA sample was more than 23 μg, and all RNA samples showed OD_260_/OD_280_ ratios ≥ 1.8 and OD_260_/OD_230_ ratios > 2.2, indicating a high purity of the extracted total RNA.

To assess the RNA quality, 150 ng of the extracted RNA sample was stained simultaneously with GelRed^®^ Nucleic Acid Stain (Sigma-Aldrich) and Orang G loading dye (Thermo Fisher Scientific) and heated to 70°C followed by cooling down on ice for 2 min, and was subsequently loaded onto wells of 1.5% agarose gels and run under 100 volts for 15 − 20 min for gel electrophoresis. All of our RNA samples showed two obvious bands of the 28S and 18S ribosomal RNAs (rRNAs) on the UV-illuminated gels without genomic DNA contamination, and the intensities of the 28S rRNA bands in all RNA samples were approximately 2-fold of the intensities of the 18S rRNA bands (data not shown), indicating a high quality of the extracted total RNA.

To further measure the RNA integrity number, Agilent RNA 6000 Nano kit was used according to the manufacturer’s instructions. Each RNA sample was assigned a score of the RNA Integrity Number (RIN), which was calculated by an algorithm taking into account the entire electrophoretic trace of the RNA sample and not only the ratio of 28S/18S rRNAs. The RIN score ranges from 1 to 10, with a RIN of 1 corresponding to a completely degraded sample carrying no apparent 28S/18S rRNA peaks, and a RIN of 10 indicating a pure, un-degraded total RNA sample with only two most prominent 28S/18S rRNA peaks. In our study, all RNA samples analyzed showed RIN scores of 10.

### Total RNA Reverse Transcription and Quantitative Real Time PCR Analyses

Two μg of each total RNA sample was reversely transcribed into single-stranded cDNAs using the Applied Biosystems^TM^ High-Capacity cDNA Reverse Transcription (RT) Kit (Thermo Fisher Scientific) according to the manufacturer’s instructions. The sizes of the PCR products and sequences of the forward and reverse primers for each gene analyzed in this study are listed in Table 2. The CFX Connect^TM^ Real-Time PCR Detection System (Bio-Rad Laboratories, Inc.) was applied to analyze each RT product sample in triplicate. The thermal cycling program was set up as 20 s of denaturation at 95°C and 39 cycles of denaturation at 95°C for 5 s followed by annealing and extension at 60°C for 30 s.

**TABLE 2:** Information of qPCR products and primers.

**Gene**	**NCBI reference sequence**	**PCR size**	**Primer name**	**Primer sequence (5′→3′)**
*BMP4*	NM_130851	90	BMP4-F	GCGTAGCCCTAAGCATCACT
	1750 bp		BMP4-R	CCCACATCGCTGAAGTCCAC
*CC10*	NM_003357	116	CC10-F	GCATTTAGAAGCTGAAGATCCCCAA
	452 bp		CC10-R	GGGCACAAAAGTGAGATGCTTGT
*FOXJ1*	NM_001454	159	FOXJ1-F	GCCAGCAAGGCCACCAAGAT
	2666 bp		FOXJ1-R	TTCGTCCTTCTCCCGAGGCA
*GAPDH*	NM_001289745	204	GAPDH-F	CATGAGAAGTATGACAACAGCCT
	1513 bp		GAPDH-R	AGTCCTTCCACGATACCAAAGT
*HEY1*	NM_012258	159	HEY1-F	TTAAAAGGGCTTTCCTGCCTCC
	2319 bp		HEY1-R	AGAGGTCAAACCCAGTTCAGTG
*HIF1A*	NM_000214	75	JAG1-F	CCTGCTGAGTCTGTTCTGGTAATCG
	5988 bp		JAG1-R	GCCTTTCAGTTCTTCCTCCATCCC
*MKI67*	NM_001145966	120	MKI67-F	CAGCACCTTTTCTCACCCTGG
	11427 bp		MKI67-R	AAACACGGGGGTAGCCCTTA
*MUC5AC*	NM_001304359	122	MUC5AC-F	AGCCATGGGAAGGTGGAGGT
	17474 bp		MUC5AC-R	TTGTCCCACAGCAGCACCAG
*NKX2-1*	NM_003317	105	NKX2-1-F	AAGGGCATAAAACAGCTTTGGG
	2352 bp		NKX2-1-R	AGAGCCATGTCAGCACAGAG
*NOTCH1*	NM_017617	113	NOTCH1-F	CGGAGTGTGTATGCCAAGAGT
	9309 bp		NOTCH1-R	TGGTTCTGGAGGGACCAAGAA
*NOTCH3*	NM_000435	106	NOTCH3-F	TGGGAGCCAGGGCAGATGTATG
	8089 bp		NOTCH3-R	GCCAGAGGATTACCAGGAAGAGAAAG
*OCT4*	NM_203289	106	POU5F1-F	GCCACACGTAGGTTCTTGAAT
	1743 bp		POU5F1-R	TGATGTCCTGGGACTGGATTT

The CFX Manager^TM^ 3.0 software (Bio-Rad Laboratories, Inc.) was used for experimental setup and data analyses. qPCR results were analyzed by the DDCt method based on the cycle threshold (Ct) values. The mean Ct value for each target mRNA was normalized against the mean Ct value for the reference mRNA of a housekeeping gene from the same RNA sample. Given that previous studies have reported *GAPDH* as one of the most stable housekeeping genes in human and rat primary cells cultured *in vitro* under hypoxia (Nagelkerke et al., 2010; Tan et al., 2012; Moein et al., 2017) and that our microarray analyses revealed less than ±0.1 in the Log2 fold changes and more than 0.5 in *P*-values in *GAPDH* gene expression in HBE cells among different oxygen tensions (data not shown), we chose *GAPDH* as the major reference gene in this study. The fold change of the expression level of each target mRNA within each experimental group or the control group was first calculated relative to the expression level of the reference *GAPDH* mRNA within the same group to obtain a value of 2^–Δ*Ct(target mRNA)*^, where ΔCt^*target mRNA*^ = Ct^*target mRNA*^ − Ct*^*GAPDH*^*^mRNA^. Subsequently, the expression level of a specific target mRNA in an experimental group was calculated in comparison with the expression level of the same mRNA in the control group to obtain the relative fold change as 2^–ΔΔ*Ct(experimental mRNA)*^, where ΔΔCt^*experimental mRNA*^ = (ΔCt^*target mRNA*^ in the experimental group) − (ΔCt^*target mRNA*^ in the control group). In our study, the relative fold change of each mRNA was calculated by comparing the expression level in the DHBE cells/tissues to the level in the NHBE cells/tissues under the same oxygen tension, and also calculated by comparing the expression level under intermittent H/R or consecutive hypoxia to the level under normoxia within the same type of HBE cells/tissues.

### Synthesis and Reverse Transfection of siRNAs

The oligonucleotides of *HIF1A* siRNA, *NKX2-1* siRNA, scrambled Stealth RNAi siRNAs, Ambion^®^ *Silencer*^®^ Select GAPDH Positive Control siRNA, and Ambion^®^ *Silencer*^®^ Negative Control No. 1 siRNA were all obtained from Thermo Fisher Scientific, Co. The sequence of the 27-mer antisense *HIF1A* siRNA was 5′-TTCTGATTTCTTCCAATTCTTCAGGdTdT-3′, corresponding to nucleotides 299−323 of the NCBI reference sequence NM_001243084. The sequence of the 27-mer antisense *NKX2-1* siRNA was 5′-TATAGCAAGGTGGAGCAGGACATGGdTdT-3′, corresponding to nucleotides 1417−1441 of the NCBI reference sequence NM_003317. The two scrambled Stealth RNAi siRNAs applied as negative controls had randomized sequences with the same nucleotide composition as in the *HIF1A* and *NKX2-1* siRNAs, respectively, with the sequence of the 27-mer antisense *HIF1A* scrambled siRNA (scrambled siRNA 1) being 5′-TTCTATTCGATTCTGCTATCTATGCdTdT-3′ and the sequence of the 27-mer antisense *NKX2-1* scrambled siRNA (scrambled siRNA 2) being 5′-TGTTGAAGGGACAAGGAATGGGACCdTdT-3′. Given that the epithelial barriers developed by well-differentiated, polarized and pseudostratified airway epithelial tissues *in vitro* have been shown to be resistant to efficient siRNA transfection (Krishnamurthy et al., 2012; Ramachandran et al., 2013), and that application of small molecule drugs inducing a more de-differentiated physiological state of the airway epithelium may affect the results of our gene expression analyses, hence we chose to conduct the procedure of “reverse transfection” of siRNAs as described previously at the time of seeding P2 HBE cells by using the Lipofectamine^TM^ RNAiMAX Transfection Reagent kit (Thermo Fisher Scientific) (Ramachandran et al., 2013). Briefly, right before seeding HBE cells onto the Millicell^®^ PTFE inserts, 75 pmole of the siRNA was diluted in 25 μl of Opti-MEM^®^ Medium and then mixed with another 25 μl of Opti-MEM^®^ Medium containing 3 μl of Lipofectamine^®^ RNAiMAX Reagent, followed by addition onto each Millicell^®^ insert. Subsequently, 2 × 10^5^ P2 HBE cells suspended in another 100 μl of Opti-MEM^®^ Medium were added into the same insert so that the final concentration of the siRNA was 500 nmol/l, and the 24-well culture plates containing these inserts were incubated at 37°C in a 5% CO_2_ incubator for 24 h. Thereafter, the Opti-MEM^®^ Medium on the apical surface of each Millicell^®^ insert was aspirated and replaced with the CnT-PR-A proliferation medium.

Once the monolayer of HBE cells have reached 100% confluence on the Millicell^®^ inserts (i.e., within 2 − 3 days after incubation in the CnT-PR-A medium), before the beginning of airway epithelial differentiation, the CnT-PR-A medium on the apical surface was aspirated and replaced with 100 μl of the Opti-MEM^®^ Medium containing 500 nmol/l of the siRNA mixed with Lipofectamine^®^ RNAiMAX Reagent again, and incubated for another 24 h at 37°C in the incubator before the Opti-MEM^®^ Medium was replaced with the CnT-PR-AD differentiation medium. In our study, *HIF1A* siRNA was transfected into both the NHBE and DHBE cells cultured under intermittent H/R, while *NKX2-1* siRNA was transfected into only the DHBE cells cultured under intermittent H/R.

### Transfection of the cDNA Clone Containing the Human *NKX2-1* Open Reading Frame (ORF)

The untagged cDNA clone containing the 1116-bp sequence of the human *NKX2-1* open reading frame (ORF) derived from the NCBI reference sequence NM_003317 and regulated by a strong cytomegalovirus (CMV) promoter and T7 promoter was obtained from OriGene Technologies, Inc. A 4.7-kb empty vector of pCMV6-XL4 was used as a negative control to exclude the possibility of any putative effects exerted by the empty vector *per se* on differentiation and/or gene expression in the ALI-cultured NHBE or DHBE tissues. The Lipofectamine^TM^ 2000 DNA Transfection Reagent kit (Thermo Fisher Scientific) was applied with the modified procedure of “reverse transfection” as described previously to enhance the transfection efficiency across the epithelial barriers formed by ALI-differentiated HBE cells (Tucker et al., 2003; Ramachandran et al., 2013). Briefly, 5 μg of *NKX2-1* cDNA and 3−4 μl of Lipofectamine^®^ 2000 Reagent were respectively mixed with two separate aliquots of 150 μl of Opti-MEM^®^ Medium in two separate 1.5-ml microcentrifuge tubes and incubated separately at room temperature for 15 min. Thereafter, the contents of these two microcentrifuge tubes were mixed together and the single tube containing *NKX2-*1 cDNA and Lipofectamine^®^ 2000 Reagent was incubated further at room temperature for another 15 min. The P2 HBE cells right after the first passage were washed three times with Opti-MEM^®^ Medium during the two 15-min incubations and then suspended in 100 μl of Opti-MEM^®^ Medium before mixing with the 300 μl mixture of *NKX2-*1 cDNA and Lipofectamine^®^ 2000 Reagent in the microcentrifuge tube, followed by seeding onto the Millicell^®^ PTFE inserts and incubation at 37°C in a 5% CO_2_ incubator for 24 h. Subsequently, the Opti-MEM^®^ Medium on the apical surface of each Millicell^®^ insert was aspirated and replaced with the CnT-PR-A proliferation medium.

Once the monolayer of HBE cells has reached 100% confluence on the Millicell^®^ inserts (i.e., within 2−3 days after incubation in the CnT-PR-A medium), before the beginning of airway epithelial differentiation, the aforementioned procedure of making a totally 300 μl mixture of 5-μg *NKX2-1* cDNA and Lipofectamine^®^ 2000 Reagent in Opti-MEM^®^ Medium was repeated again, and CnT-PR-A medium on the apical surface of each Millicell^®^ insert was aspirated and replaced with the 300-μl mixture of the cDNA-lipid complex, followed by incubation for another 24 h at 37°C in the incubator before replacing the Opti-MEM^®^ Medium mixture with the CnT-PR-AD differentiation medium. In our study, *NKX2-1* cDNA was transfected into both the NHBE and DHBE cells cultured under consecutive hypoxia.

### Statistical Analysis

In this study, all values of the immunofluorescence staining intensity and mRNA expression levels were statistically analyzed by two-way analysis of variance (ANOVA) using GraphPad Prism 8.0 software, with the confidence interval set up as 95% (i.e., α < 0.05 or *p* < 0.05) and the multiple comparison test set up as Bonferroni’s test. For immunofluorescence staining, four cryovials for each of the P2 NHBE1, NHBE2, NHBE3, DHBE1, DHBE2, and DHBE3 cell lines were independently thawed, cultured and analyzed (*n* = 4). For real-time qPCR analyses, three cryovials for each of the P2 NHBE1, NHBE2, NHBE3, DHBE1, DHBE2, and DHBE3 cell lines were independently thawed, cultured and analyzed (*n* = 3). The mean values ± SD are shown in all of the statistical charts presented in this study.

## Results

### Consecutive Hypoxia and Intermittent H/R Differentially Affect Expression of the Ciliated and Goblet Cell-Specific Marker Proteins in Normal and COPD-Diseased HBE Cells

**FIGURE 1:**
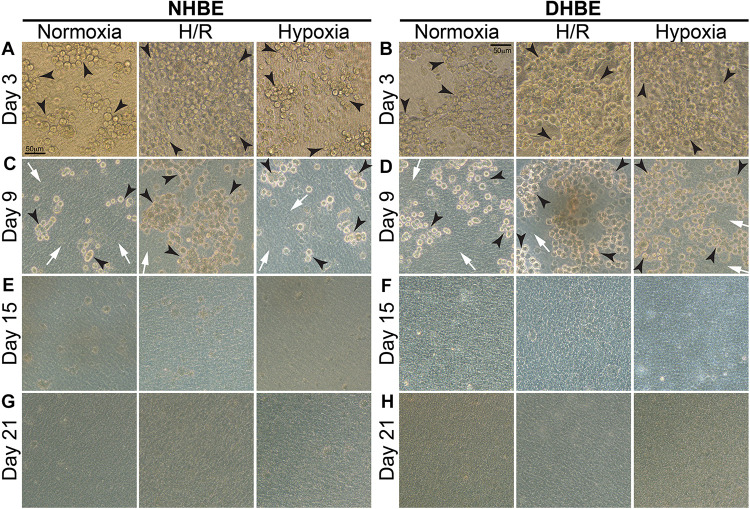
Light microscope images of the P2 HBE cells grown in Millicell^®^ PTFE inserts during the initial 3 days of proliferation and the following 18 days of ALI-induced differentiation. **(A,B)** During culturing in the CnT-PR-A medium at the proliferative stage, apparent clustering of spheric cells was observed on the top of the confluent monolayer of HBE cells at Passage 2 (P2), with the numbers of spheric cells markedly increased in the NHBE cells cultured under intermittent H/R and in the DHBE cells cultured under both intermittent H/R and consecutive hypoxia (indicated by black arrowheads). **(C,D)** After 6 days of ALI culturing, most HBE cells on the bottom layer have turned into a blue-gray color and become more flattened (indicated by white arrows), indicating early differentiation, while there were still clusters of spheric HBE cells on the top layer, with the greatest numbers of spheric cells present in the NHBE tissues cultured under intermittent H/R and in the DHBE tissues cultured under both intermittent H/R and consecutive hypoxia (indicated by black arrowheads). **(E,F)** The numbers of the spheric HBE cell clusters have significantly decreased on the top layer and most HBE cells have become embedded into the flattened bottom layer since the 12th day of ALI culturing (i.e., Day 15 of ALI culturing). **(G,H)** On Day 21 of ALI culturing, there was no more visible cluster of spheric HBE cells and the whole HBE tissues have become even more densely packed, indicating almost complete differentiation of both the NHBE and DHBE tissues. The scale bar in **(A)** represents 50 μm and applies to **(A–H)**.

Our observation using light microscopy indicated that both NHBE and DHBE cells reached confluence after 3 days of submerged culture (i.e., Day 3) on the Millicell^®^ hydrophilic polytetrafluoroethylene (PTFE) transmembrane inserts (Merck Millipore, Burlington, MA, United States) in the CnT Prime Airway proliferation medium CnT-PR-A (CELLnTEC Advanced Cell Systems AG, Bern, Switzerland) (Figures 1A,B). Interestingly, while intermittent H/R exposure caused dramatic increases in the amounts of spheric cell clusters on the top layer in both the NHBE and DHBE cultures, consecutive exposure to 1% O_2_ led to significantly increased numbers of spheric cell clusters in only the DHBE but not NHBE culture (indicated by arrowheads in Figures 1A,B). After replacement of the CnT-PR-A proliferation medium with the CnT Prime Airway Differentiation (CnT-PR-AD) medium on Day 4 followed by 5 days of ALI culturing, on Day 9 the bottom layer of HBE cells have become flattened and turned into blue-gray color (indicated by white arrows in Figures 1C,D), which suggested the initiation of ALI-induced differentiation, though the upper layer of HBE cells still remained spheric and clustered as seen on the third day (indicated by black arrowheads in Figures 1C,D). Starting from Day 15, which was on the 12th day of ALI culturing, the amount of spheric HBE cells on the top layer markedly decreased, while most cells have become flattened and embedded into the blue-gray bottom layer (Figures 1E,F). On Day 21, which was after 17 days of ALI culturing, there were no more visible spheric HBE cells clustering on the top layer (Figures 1G,H), suggesting almost complete differentiation of both NHBE and DHBE cells after totally 20 days of ALI culturing, which included the first 3 days of the proliferative stage.

**FIGURE 2:**
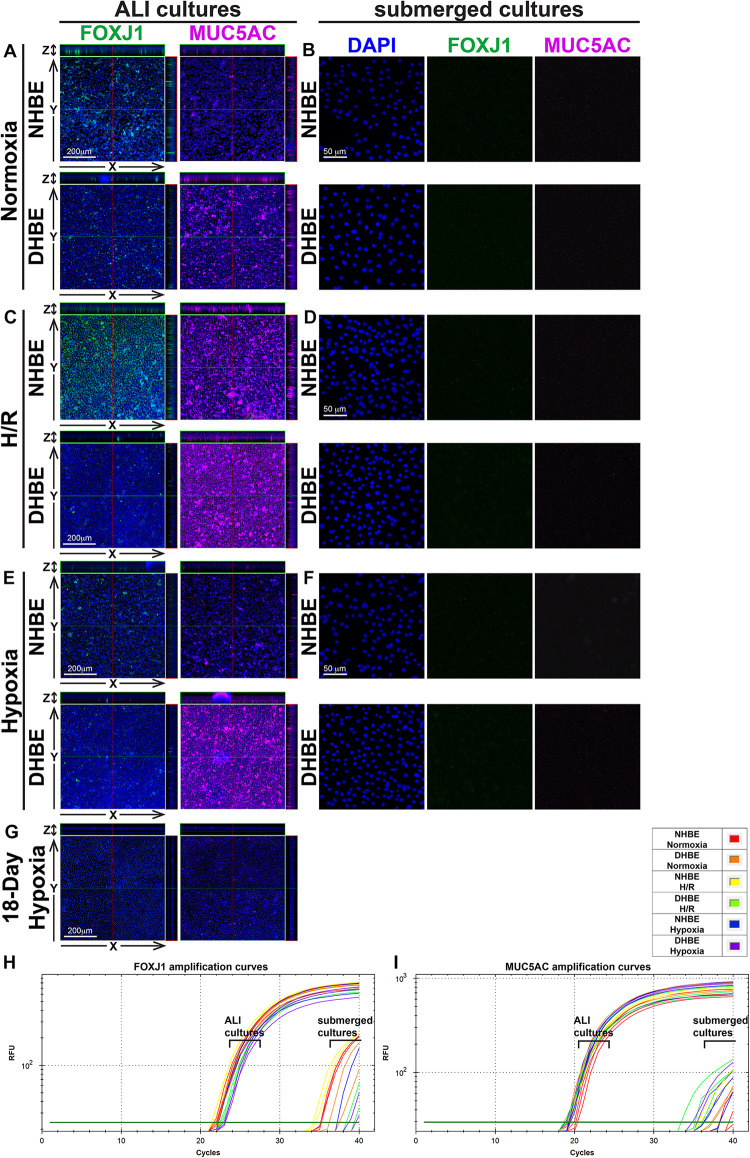
Intermittent H/R and consecutive hypoxia significantly altered immunofluorescence intensities of the ciliated cell transcription factor FOXJ1 and the goblet cell mucin protein MUC5AC in the ALI-cultured and differentiated HBE tissues. **(A,C,E,G)** Are representative immunostaining images of selected sectioning planes of the differentiated HBE tissues in the orthogonal view after ALI culturing, while **(B,D,F)** are representative immunostaining images of the undifferentiated HBE cells in the submerged cultures. The scale bars in **(A,C,E)** all represent 200 μm and the scale bars in **(B,D,F)** all represent 50 μm. **(H,I)** Show the qPCR amplification curves of FOXJ1 and MUC5AC mRNAs respectively extracted from the NHBE and DHBE cells in the ALI and submerged cultures.

Our double immunofluorescence staining revealed only background levels of both the FOXJ1 (green) and MUC5AC (magenta) immunofluorescence signals in both the NHBE and DHBE cells in the submerged cultures, and there was no significant difference in the immunofluorescence intensity between normoxia, intermittent H/R and consecutive hypoxia (Figures 2B,D,F). On the other hand, after 18 days of ALI culture under normoxia, the differentiated NHBE tissue expressed a significantly higher level of the ciliated cell marker FOXJ1, which is a major transcription factor inducing ciliogenesis (You et al., 2004; Yu et al., 2008), and a lower level of the goblet cell marker MUC5AC, which is a predominant secreted airway mucin (Hovenberg et al., 1996; Lillehoj et al., 2013), whereas the differentiated DHBE tissue expressed a significantly lower level of FOXJ1 and a higher level of MUC5AC (Figure 2A). Under normoxia, the percentages of FOXJ1^+^ and MUC5AC^+^ cells in the differentiated NHBE tissues were respectively 34.84 ± 7.19% and 16.82 ± 6.47% (*n* = 12 with all three different groups of NHBE tissues averaged together, see also Supplementary Figures S1A,B), in agreement with the previously demonstrated constitution of 20–30% of ciliated cells and 15–20% of goblet cells in the ALI-induced differentiated human airway epithelial cells (Schamberger et al., 2015; Benam et al., 2016). On the other hand, the percentages of FOXJ1^+^ and MUC5AC^+^ cells in the differentiated DHBE tissues were respectively 10.82 ± 2.95% and 31.91 ± 9.25% (*n* = 12 with all three different groups of DHBE tissues averaged together, see also Supplementary Figures S1A,B), which showed respectively more than 66% decreases and 1.8-fold increases compared to the percentages in the differentiated NHBE tissues.

After 18 days of ALI culture under intermittent 24/24-h cycles of H/R, the expression levels of both FOXJ1 and MUC5AC proteins in the differentiated NHBE tissues were markedly increased (compare Figure 2C with Figure 2A). The average percentages of FOXJ1^+^ and MUC5AC^+^ cells in the NHBE tissues cultured under intermittent H/R, which were respectively 72.54 and 57.46%, were respectively more than two and three times the average percentages in the NHBE tissues cultured under normoxia (Supplementary Figures S1A,B). On the other hand, in the differentiated DHBE tissues, intermittent H/R caused an almost 2-fold increase in the average percentage of MUC5AC^+^ cells whereas a more than 50% decrease in the average percentage of FOXJ1^+^ cells compared to normoxia (Figure 2C and Supplementary Figures S1A,B), indicating that intermittent H/R stimulated MUC5AC expression whereas exerted opposite effects on FOXJ1 expression in NHBE and DHBE cells during ALI-induced differentiation.

During differentiation induction by ALI culturing, in contrast to the opposite effects of intermittent H/R on FOXJ1 expression in NHBE and DHBE tissues, consecutive hypoxia exerted suppressive effects on FOXJ1 expression in both NHBE and DHBE tissues. After 9 days of ALI culture under consecutive hypoxia (1% O_2_) followed by 9 days of ALI culture under normoxia, the percentages of FOXJ1^+^ cells in the differentiated NHBE and DHBE tissues showed respectively more than 50% and more than 60% decreases after 9 days of ALI culture under consecutive hypoxia (1% O_2_) followed by 9 days of ALI culture under normoxia (Figure 2E and Supplementary Figure S1A). On the other hand, as compared to normoxia, 9-day consecutive hypoxia led to an over 2-fold increase in the average percentage of MUC5AC^+^ cells in the DHBE tissues (i.e., 64.65 ± 12.98% vs. 31.91 ± 9.25%) without significantly affecting the percentages of MUC5AC^+^ cells in the NHBE tissues (i.e., 16.82 ± 6.47% vs. 15.34 ± 2.89%) (Supplementary Figure S1B). Interestingly, NHBE3 cells, which were derived from a healthy donor with no smoking history and no cardiovascular disease, showed the highest average percentages of FOXJ1^+^ cells and the lowest average percentages of MUC5AC + cells among all six groups of HBE cells in the ALI cultures under normoxia, intermittent H/R and 9-day consecutive hypoxia (green labels in Supplementary Figures S1A,B). It is noteworthy that ALI culture under consecutive hypoxia for 18 days significantly inhibited normal differentiation of both NHBE and DHBE cells, as the percentages of FOXJ1^+^ and MUC5AC^+^ cells both dramatically reduced to less than 10% of the percentages in NHBE and DHBE tissues cultured under normoxia (Figure 2G and Supplementary Figures S1A,B). Because the differentiation capacities of both NHBE and DHBE cells were dramatically suppressed after ALI culturing under consecutive hypoxia for 18 days, the subsequent analyses of mucociliary gene and protein expression were focused on intermittent H/R and consecutive hypoxia for 9 days only.

In agreement with the immunostaining analyses, our qPCR analyses also revealed significantly decreased levels of *FOXJ1* mRNA and increased levels of *MUC5AC* mRNA in DHBE cells compared to NHBE cells under normoxia in both the ALI and submerged cultures (Figures 2H,I and Supplementary Figures S1C,D). In addition, intermittent H/R significantly increased both *FOXJ1* and *MUC5AC* mRNA levels in the ALI-cultured NHBE cells, while intermittent H/R and consecutive hypoxia both significantly decreased the level of *FOXJ1* mRNA and increased the level of *MUC5AC* mRNA in the ALI-cultured DHBE cells, same as observed in the immunostaining analyses (Figures 2H,I and Supplementary Figures S1C,D). The highest *FOXJ1* mRNA levels and lowest *MUC5AC* mRNA levels under normoxia, intermittent H/R and 9-day consecutive hypoxia among all six groups of HBE cells were also observed in NHBE3 cells (green labels in Supplementary Figures S1C,D). Therefore, both intermittent H/R and consecutive hypoxia affect FOXJ1 and MUC5AC expression in the HBE cells at both the mRNA and protein levels.

### Intermittent H/R and Consecutive Hypoxia Exert Differential Effects on Apoptotic FOXJ1^+^ Cells and Proliferating MUC5AC^+^ Cells in the Differentiated NHBE and DHBE Tissues

As we found that intermittent H/R exerted the same stimulatory effects on both the protein and mRNA levels of FOXJ1 and MUC5AC in the differentiating NHBE cells (Figures 2C,H,I and Supplementary Figure S1), whereas both intermittent H/R and consecutive hypoxia exerted opposite effects on the protein and mRNA levels of FOXJ1 and MUC5AC (i.e., inhibitory effect on the FOXJ1 level and stimulatory effect on the MUC5AC level) in the differentiating DHBE cells (Figures 2E,H,I and Supplementary Figure S1), we were interested in further deciphering whether the differential effects resulted from distinct influences of intermittent H/R and consecutive hypoxia on the apoptosis and proliferation rates of NHBE and DHBE cells. Therefore, we conducted double immunostaining of active Caspase-3 and FOXJ1 to analyze whether the decreased immunofluorescence intensities of FOXJ1 in the DHBE tissues were associated with increased apoptosis of FOXJ1-expressing cells, and we performed double immunostaining of Ki67 and MUC5AC to analyze whether the increased immunofluorescence intensities of MUC5AC in the DHBE tissues were associated with increased proliferation of MUC5AC-expressing cells.

**FIGURE 3:**
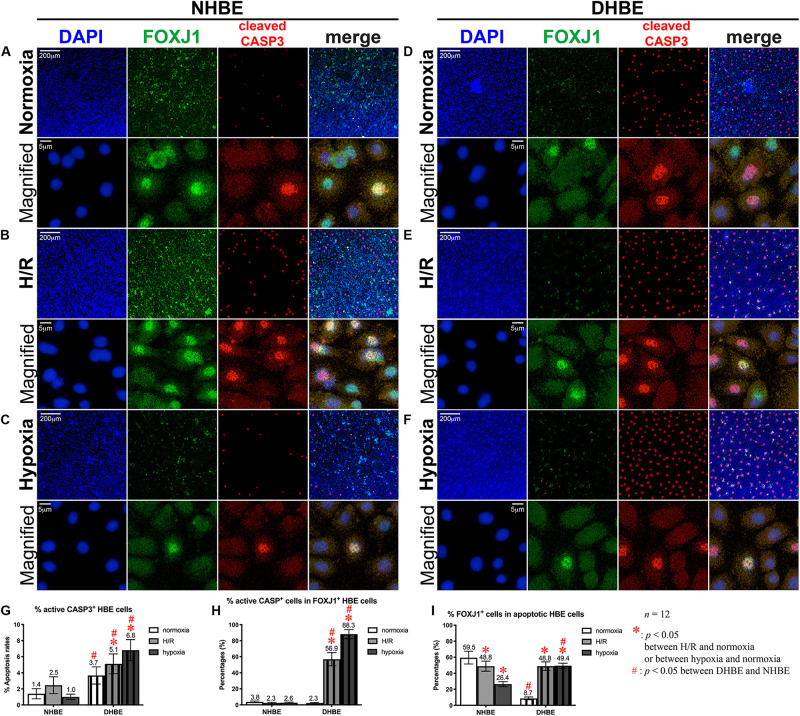
Both intermittent H/R and consecutive hypoxia significantly increased the apoptotic rates in both the whole tissue and within the FOXJ1-expressing cell population in the differentiated DHBE cultures. **(A–C)** Double immunofluorescence staining in NHBE cells in ALI cultures showed partial colocalization of FOXJ1 (green) and cleaved caspase-3 (red) in the same nuclei. **(D–F)** Double immunofluorescence staining in DHBE cells in ALI cultures showed partial colocalization of FOXJ1 (green) and cleaved caspase-3 (red) in the same nuclei. The scale bars in **(A,D)** both represent 200 μm and apply to **(A–F)** except the magnified panels, in which the scale bars represent 5 μm. **(G–I)** Statistical charts showing the respective percentages of cleaved caspase-3^+^ nuclei and colocalized FOXJ1^+^ and apoptotic nuclei in both the NHBE and DHBE cells in the ALI cultures.

**FIGURE 4:**
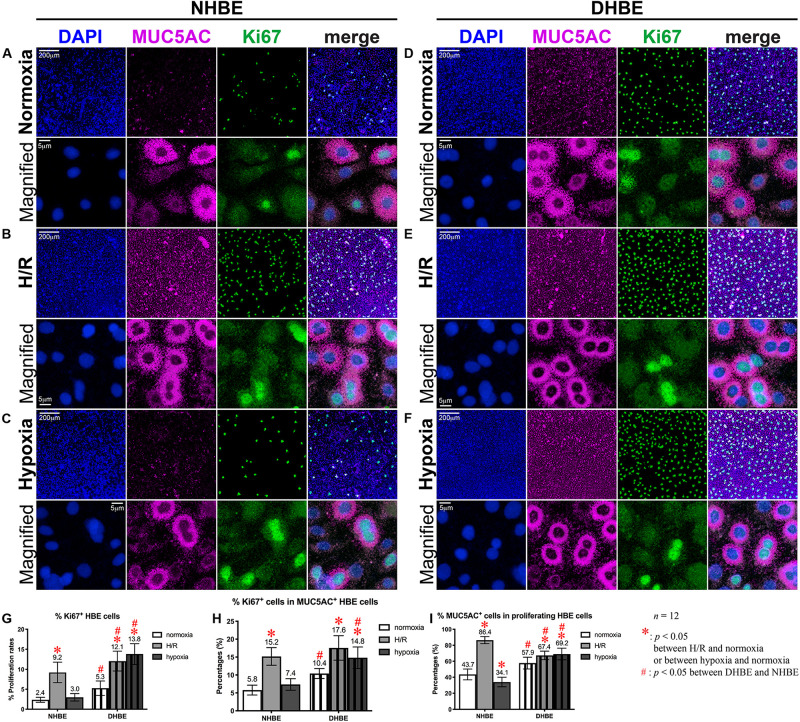
Both intermittent H/R and consecutive hypoxia significantly increased the proliferation rates in both the whole tissue and within the MUC5AC-expressing cell population in the differentiated DHBE cultures. **(A–C)** Double immunofluorescence staining in NHBE cells in ALI cultures showed partial colocalization of MUC5AC (magenta) and Ki67 (green) in the same cells. **(D–F)** Double immunofluorescence staining in DHBE cells in ALI cultures showed partial colocalization of MUC5AC (magenta) and Ki67 (green) in the same cells. The scale bars in **(A,D)** both represent 200 μm and apply to panels **(A–F)** except the magnified panels, in which the scale bars represent 5 μm. **(G–I)** Statistical charts showing the respective percentages of Ki67^+^ nuclei and colocalized MUC5AC^+^ and proliferating cells in both the NHBE and DHBE cells in the ALI cultures.

After culturing under normoxia for 18 days, in comparison with the NHBE tissues, the average percentage of total active Caspase-3^+^ cells was significantly increased in the DHBE tissues (i.e., a more than 2.5-fold increase in Figure 3G, *p* < 0.05, two-way ANOVA), while the average percentages of active Caspase-3^+^ cells within the FOXJ1^+^ cell population were comparable between the DHBE and NHBE tissues (i.e., no significant change in Figure 3H), and the average percentage of FOXJ1^+^ cells within the apoptotic cell population was significantly decreased in the DHBE tissues (i.e., a more than 85% decrease in Figure 3I, *p* < 0.05, two-way ANOVA). On the other hand, in comparison with the NHBE tissues cultured under normoxia, the average percentage of total Ki67^+^ cells, the percentage of Ki67^+^ cells within the MUC5AC^+^ cell population, and the percentage of MUC5AC^+^ cells within the proliferating cell population were all significantly and consistently higher in the DHBE tissues compared to the NHBE tissues (i.e., more than 1.3-fold increases in Figures 4G–I, *p* < 0.05, two-way ANOVA). Interestingly, we found that intermittent H/R markedly increased the proliferation rate without significantly affecting the apoptosis rate of NHBE cells, whereas both intermittent H/R and 9-day consecutive hypoxia significantly increased both the apoptosis and proliferation rates of DHBE cells (Figures 3B,C,E–G, 4B,C,E–G).

Within the populations of FOXJ1^+^ cells, while both intermittent H/R and consecutive hypoxia significantly increased active Caspase-3-positive immunostaining signals in DHBE cells, there was no significant change in active Caspase-3-positive signals in NHBE cells after ALI culture under intermittent H/R or 9-day consecutive hypoxia (Figure 3H). Within the populations of active Caspase-3^+^ apoptotic cells, both intermittent H/R and consecutive hypoxia markedly decreased FOXJ1-positive immunostaining signals in NHBE cells, in contrast to the dramatic increases in FOXJ1-positive signals in DHBE cells (Figure 3I). Under normoxia, the average apoptosis rate in the DHBE tissues (3.67 ± 1.06%) showed a more than 2.5-fold increase compared to the average apoptosis rate in the NHBE tissues (1.40 ± 0.63%) (compare Figures 3A–D, and refer to Figure 3G), whereas the average percentage of FOXJ1^+^ cells within the apoptotic cell population in the DHBE tissues (8.66 ± 1.81%) showed a more than 85% decrease compared to the average percentage in the NHBE tissues (59.50 ± 7.94%) (compare Figures 3A–D, and refer to Figure 3I), indicating that, within the apoptotic cell populations, there were much fewer FOXJ1-expressing cells in the DHBE tissues compared to the NHBE tissues under normoxia.

It is noteworthy that ALI culture under intermittent H/R led to similar percentages of FOXJ1-expressing cells within the apoptotic cell populations between the differentiated NHBE and DHBE tissues (i.e., 48.83 ± 6.27% vs. 48.84 ± 5.38%) (compare Figures 3B–E, and refer to Figure 3I). ALI culture under 9 days of consecutive hypoxia followed by 9 days of normoxia further decreased the average percentage of FOXJ1-expressing cells in the apoptotic NHBE cell populations to less than 55% of the average percentage in the apoptotic DHBE cell populations (compare Figures 3C–F, and refer to Figure 3I). As the total apoptosis rate in the differentiated NHBE tissues was not significantly affected by intermittent H/R or consecutive hypoxia (compare Figures 3A–C, and refer to Figure 3G), within the apoptotic NHBE cell population the marked decrease in the percentage of FOXJ1^+^ cells induced by intermittent H/R and consecutive hypoxia (Figure 3I) was plausibly compensated by an increased percentage of non-FOXJ1-expressing cells.

In contrast to the differential effects of intermittent H/R on the changes of the apoptosis rates and percentages of FOXJ1^+^ cells in the NHBE versus DHBE tissues (Figures 3G–I), ALI culture under intermittent H/R consistently increased the total proliferation rates (Figures 4B,E,G), and increased the proliferation rates within the MUC5AC^+^ cell populations (Figure 4H), as well as increased the percentages of MUC5AC^+^ cells within the proliferating cell populations (Figure 4I) in both the differentiated NHBE and DHBE tissues. On the other hand, ALI culture under consecutive hypoxia for 9 days followed by normoxia for another 9 days significantly increased the total proliferation rate and the proliferation rate within the MUC5AC^+^ cell population in the DHBE but not NHBE tissues (Figures 4G,H).

As for the percentages of MUC5AC^+^ cells within the proliferating cell populations, 9 days of consecutive hypoxia significantly increased the average percentage in the DHBE tissues whereas significantly decreased the average percentage in the NHBE tissues (Figure 4I), indicating that consecutive hypoxia exerted opposite effects on the predominance of MUC5AC-expressing cells within the proliferating cell populations in the differentiated NHBE and DHBE tissues.

### Concordant and Discordant Changes in mRNA Expression Levels Induced by Intermittent H/R or Consecutive Hypoxia Identify Distinct Gene Modules Regulating MUC5AC and FOXJ1 Expression in Differentiated HBE Tissues

**FIGURE 5:**
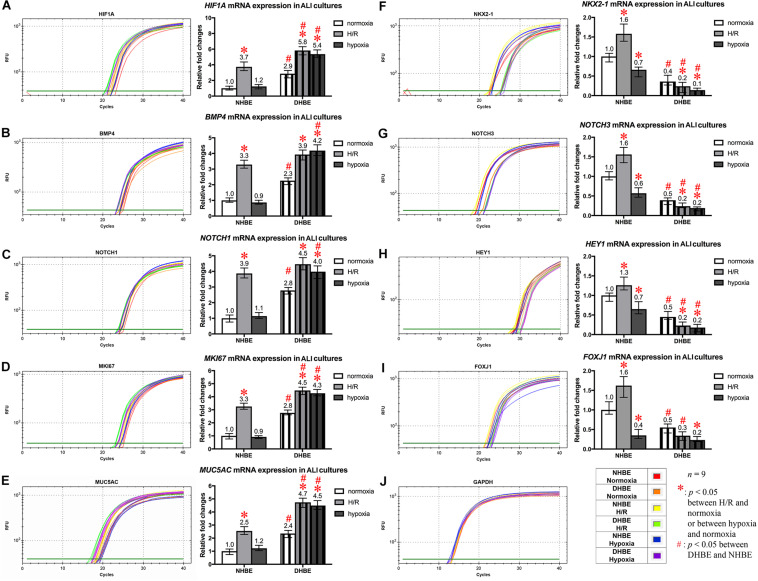
Concordant regulation of *HIF1A*, *BMP4*, *NOTCH1*, *MKI67*, and *MUC5AC* mRNA expression and concordant regulation of *NKX2-1*, *NOTCH3*, *HEY1*, and *FOXJ1* mRNA expression by intermittent H/R and consecutive hypoxia in the NHBE and DHBE tissues. Among several candidate genes in our preliminary microarray analyses (data not shown) whose expression levels were regulated concordantly with the levels of *MUC5AC* and *FOXJ1*, respectively, in the HBE cells cultured under intermittent H/R or consecutive hypoxia, *HIF1A*, *BMP4*, *NOTCH1*, and *MKI67* mRNA levels were concordantly upregulated with the level of *MUC5AC* mRNA by intermittent H/R in both the differentiated NHBE and DHBE tissues (**A–E**: *n* = 12, *p* < 0.001), while *NKX2-1*, *NOTCH3*, and *HEY1* mRNA levels were concordantly downregulated with the level of *FOXJ1* mRNA by consecutive hypoxia in both the differentiated NHBE and DHBE tissues (**F–J**: *n* = 12, *p* < 0.05). In addition, the expression levels of *HIF1A*, *BMP4*, *NOTCH1*, *MKI67*, and *MUC5AC* were consistently higher in the DHBE tissues compared to the NHBE tissues under all culturing conditions (**A–E**: *n* = 12, *p* < 0.05 except for *BMP4* and *NOTCH1* mRNA levels under intermittent H/R, which showed *p* > 0.99 when compared between the NHBE and DHBE tissues), while the expression levels of *NKX2-1*, *NOTCH3*, *HEY1*, and *FOXJ1* were consistently lower in the DHBE tissues compared to the NHBE tissues under all culturing conditions (**F–J**: *n* = 12, *p* < 0.001 except for FOXJ1 mRNA levels under consecutive hypoxia, which showed *p* = 0.18 when compared between the NHBE and DHBE tissues). On the other hand, consecutive hypoxia led to concordant upregulation of *HIF1A*, *BMP4*, *NOTCH1*, *MKI67*, and *MUC5AC* mRNA levels in the DHBE tissues but not the NHBE tissues (**A–E**: *n* = 12, *p* < 0.001), while intermittent H/R led to concordant upregulation of *NKX2-1*, *NOTCH3*, *HEY1*, and *FOXJ1* mRNA levels in the NHBE tissues but not the DHBE tissues (**F–J**: *n* = 12, *p* < 0.05). The statistical analysis applied in this figure has a mean square value between groups as 5 and a mean square value within groups as 6, hence the *F* statistic is 4.39. The *F*-values calculated from our data for this figure are all greater than 4.39 and thus the null hypothesis is rejected. The asterisks (^∗^) indicate *p* < 0.05 when comparing the HBE cells cultured under intermittent H/R or consecutive hypoxia with the same type of cells cultured under normoxia, and the hashtags (#) indicate *p* < 0.05 when comparing the DHBE tissues with the NHBE tissues cultured under the same oxygen tension.

To further unravel the molecular mechanisms underlying the differential regulation of FOXJ1 and MUC5AC expression by intermittent H/R and consecutive hypoxia in the differentiated NHBE and DHBE tissues, we performed quantitative polymerase chain reactions (qPCR) to quantify the mRNA levels of the hypoxia-inducible factors, lung stem/progenitor cell markers, and signaling factors regulating lung lineage specification/differentiation. Among the genes analyzed in both the NHBE and DHBE tissues after ALI culture under intermittent H/R or consecutive hypoxia, we found that the changes in the expression levels of *HIF1A*, *BMP4*, *NOTCH1*, *MKI67* and *MUC5AC* mRNAs were concordant with each other (Figures 5A–E), while the changes in the expression levels of *NKX2-1*, *NOTCH3*, *HEY1*, and *FOXJ1* mRNAs were concordant with each other (Figures 5F–I). The mRNA levels of *HIF1A*, *BMP4*, *NOTCH1*, *MKI67*, and *MUC5AC* were significantly and concordantly increased by both intermittent H/R and consecutive hypoxia in the DHBE tissues whereas increased by only intermittent H/R rather than consecutive hypoxia in the NHBE tissues (Figures 5A–E). On the other hand, the mRNA levels of *NKX2-1*, *NOTCH3*, *HEY1*, and *FOXJ1* were significantly and concordantly decreased by both intermittent H/R and consecutive hypoxia in the DHBE tissues whereas increased by intermittent H/R and decreased by consecutive hypoxia in the NHBE tissues (Figures 5F–I). For each group of the NHBE and DHBE cells, three independent experiments of mRNA extraction and qPCR analyses were performed, and *HIF1A*, *BMP4*, *NOTCH1*, *MKI67*, *MUC5AC*, *NKX2-1*, *NOTCH3*, *HEY1*, and *FOXJ1* mRNAs all exhibited consistent changes in the increases or decreases of the amplification cycle numbers among the three independent qPCR analyses when compared between the six groups of HBE cells analyzed (Supplementary Figures S2, S3).

**FIGURE 6:**
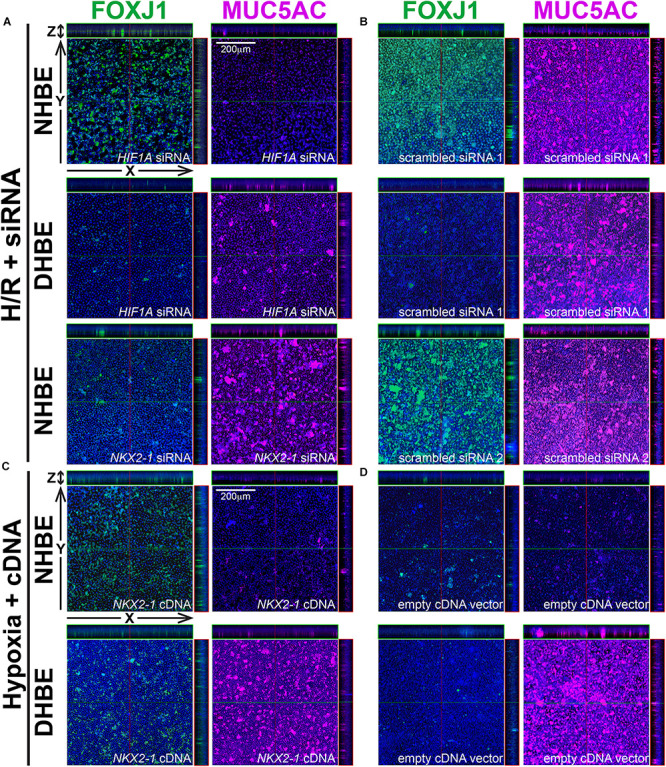
In ALI-cultured HBE tissues, transfection with *HIF1A* or *NKX2-1* siRNA significantly decreased the immunofluorescence intensity of MUC5AC or FOXJ1 under intermittent H/R, while transfection with *NKX2-1* cDNA significantly increased the immunofluorescence intensity of FOXJ1 under consecutive hypoxia. **(A–D)** Representative immunofluorescence staining images of selected Z-stack slice images of the differentiated NHBE and DHBE tissues in the orthogonal view after ALI culturing. The images in **(A)** are from *HIF1A* or *NKX2-1* siRNA-transfected NHBE and DHBE cells cultured under intermittent H/R, while the images in **(B)** are from scrambled siRNA-transfected NHBE and DHBE cells cultured under intermittent H/R. The images in **(C)** are from *NKX2-1* cDNA-transfected NHBE and DHBE cells cultured under 9-day consecutive hypoxia, while the images in **(D)** are from empty cDNA vector-transfected NHBE and DHBE cells cultured under consecutive hypoxia. The scale bars in **(A–D)** represent 200 μm.

Because our results of qPCR analyses indicated that the changes in the expression levels of *HIF1A* and *MUC5AC* mRNAs were concordant with each other, while those of *NKX2-1* and *FOXJ1* mRNAs were concordant with each other, we were interested in assessing whether modulation of *HIF1A* or *NKX2-1* expression could respectively affect expression of the prominent airway mucin gene *MUC5AC* or the ciliogenesis-inducing transcription factor gene *FOXJ1* in the differentiated HBE tissues. *HIF1A* siRNA, *NKX2-1* siRNA or *NKX2-1* cDNA were respectively transfected into the NHBE and DHBE cells right after subculturing and seeding onto the Millicell^®^ PTFE transmembrane inserts (see section Materials and Methods). To analyze the efficiency of *HIF1A* gene knockdown, our qPCR analyses of *HIF1A* mRNA expression revealed an approximately 70% decrease in the *HIF1A* mRNA level within each group of the *HIF1A* siRNA-transfected NHBE cells (e.g., NHBE1, NHBE2, and NHBE3) cultured under normoxia and more than 85% decreases in the *HIF1A* mRNA levels in all three groups of the *HIF1A* siRNA-transfected DHBE cells (e.g., DHBE1, DHBE2, and DHBE3) cultured under normoxia (Supplementary Figures S4A, S5A). Interestingly, under intermittent H/R, *HIF1A* siRNA transfection dramatically caused more than 98% decreases in the *HIF1A* mRNA levels in all three groups of both NHBE and DHBE cells (indicated by triplet asterisks in Supplementary Figure S1B), indicating that the gene knockdown efficiency of *HIF1A* siRNA was significantly greater under intermittent H/R compared to normoxia (Supplementary Figures S4A, S5A). Our immunostaining analyses revealed that, after 18 days of ALI culture, *HIF1A* siRNA transfection significantly reduced the intermittent H/R-increased percentages of MUC5AC^+^ cells in both the NHBE and DHBE tissues to approximately the same levels under normoxia without significantly affecting the percentages of FOXJ1^+^ cells (compare Figure 6A with Figure 6B, and see also Supplementary Figures S6A,B).

Similar to the phenomenon of increased gene knockdown efficiency of *HIF1A* siRNA under intermittent H/R, the efficiency of gene knockdown by *NKX2-1* siRNA was also higher in the NHBE cells cultured under intermittent H/R (causing more than 69% decreases in the *NKX2-1* mRNA levels) compared to those cultured under normoxia (causing more than 62% decreases in the *NKX2-1* mRNA levels) (Supplementary Figures S4B, S5B). We found that *NKX2-1* siRNA transfection significantly reduced the intermittent H/R-increased percentages of FOXJ1^+^ cells in the NHBE tissues to approximately the same levels under normoxia without significantly affecting the percentages of MUC5AC^+^ cells (compare Figure 6A with Figure 6B). Consistent with the concordant changes in *NKX2-1* and FOXJ1 expression in the differentiated HBE tissues, we also found that *NKX2-1* overexpression by cDNA transfection significantly caused over 4.5-fold increases in the percentages of FOXJ1^+^ cells without affecting the percentages of MUC5AC^+^ cells in both the NHBE and DHBE tissues cultured under consecutive hypoxia (compare Figure 6C with Figure 6D, and see also Supplementary Figures S6C,D). Our qPCR analyses revealed that, under normoxia, *NKX2-1* cDNA transfection significantly caused more than 3-fold increases in the *NKX2-1* mRNA levels in NHBE cells and caused more than 13-fold increases in the *NKX2-1* mRNA levels in DHBE cells, while under hypoxia, *NKX2-1* cDNA transfection significantly caused more than 3-fold increases in the *NKX2-1* mRNA levels in NHBE cells and caused approximately 5- to 9-fold increases in the *NKX2-1* mRNA levels in DHBE cells (Supplementary Figure S5C).

**FIGURE 7:**
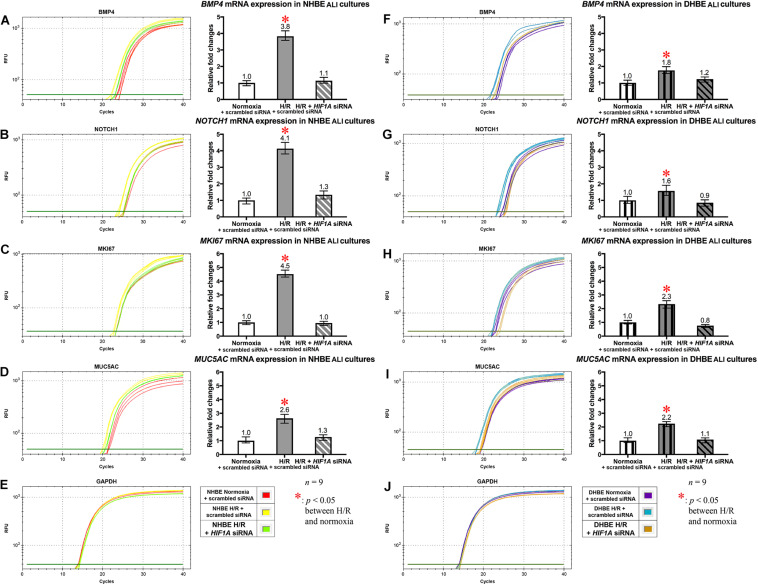
*HIF1A* siRNA transfection concordantly downregulated the mRNA levels of *BMP4*, *NOTCH1*, *MKI67*, and *MUC5AC* in both the differentiated NHBE and DHBE tissues. Transfection of *HIF1A* siRNA into both the NHBE and DHBE cells followed by ALI culturing under intermittent H/R consistently and significantly decreased the mRNA levels of *BMP4*, *NOTCH1*, *MKI67*, and *MUC5AC* in both the differentiated NHBE **(A–D)** and DHBE **(F–I)** tissues compared to the levels in both the NHBE and DHBE tissues transfected with the scrambled negative control siRNA and cultured under intermittent H/R [which were indicated by asterisks in **(A–D,F–I)**] (*n* = 12, *p* < 0.001 for the NHBE tissues and *p* < 0.05 for the DHBE tissues). Therefore, after *HIF1A* siRNA transfection, the mRNA levels of *BMP4*, *NOTCH1*, *MKI67*, and *MUC5AC* in both the NHBE and DHBE tissues cultured under intermittent H/R became comparable with the levels in the NHBE and DHBE tissues cultured under normoxia (*n* = 12, *p* > 0.99). The asterisks (^∗^) indicate *p* < 0.05 when comparing the HBE cells cultured under intermittent H/R with the same type of cells cultured under normoxia. **(E,J)** qPCR amplification curves of the mRNA levels of the reference gene GAPDH in NHBE and DHBE tissues, respectively.

In addition to the immunostaining analyses of the mucous and ciliated lineage-specific markers MUC5AC and FOXJ1 in the differentiated HBE cells, we also performed qPCR analyses to further assess whether *BMP4*, *NOTCH1*, *MKI67*, and *MUC5AC* mRNAs were all co-regulated by *HIF1A* expression, and whether *NOTCH3*, *HEY1*, and *FOXJ1* mRNAs were all co-regulated by *NKX2-1* expression. In the *HIF1A* siRNA-transfected and ALI-cultured NHBE and DHBE tissues after 18 days of intermittent H/R, the expression levels of *BMP4*, *NOTCH1*, *MKI67*, and *MUC5AC* mRNAs all decreased to approximately the same as those in the NHBE and DHBE tissues cultured under normoxia for 18 days (Figures 7A−D,F−I]). For each group of the NHBE and DHBE cells cultured under normoxia and H/R, three independent experiments of *HIF1A* or scrambled siRNA transfection and qPCR analyses were performed, and *BMP4*, *NOTCH1*, *MKI67*, and *MUC5AC* mRNAs all exhibited consistent changes in the increases or decreases of the amplification cycle numbers among the three independent qPCR analyses when compared between different groups of HBE cells analyzed (Supplementary Figure S7). Hence our results revealed that the mRNA levels of *BMP4*, *NOTCH1*, *MKI67* and *MUC5AC* were all co-upregulated by *HIF1A* during ALI-induced differentiation of HBE cells under intermittent H/R.

**FIGURE 8:**
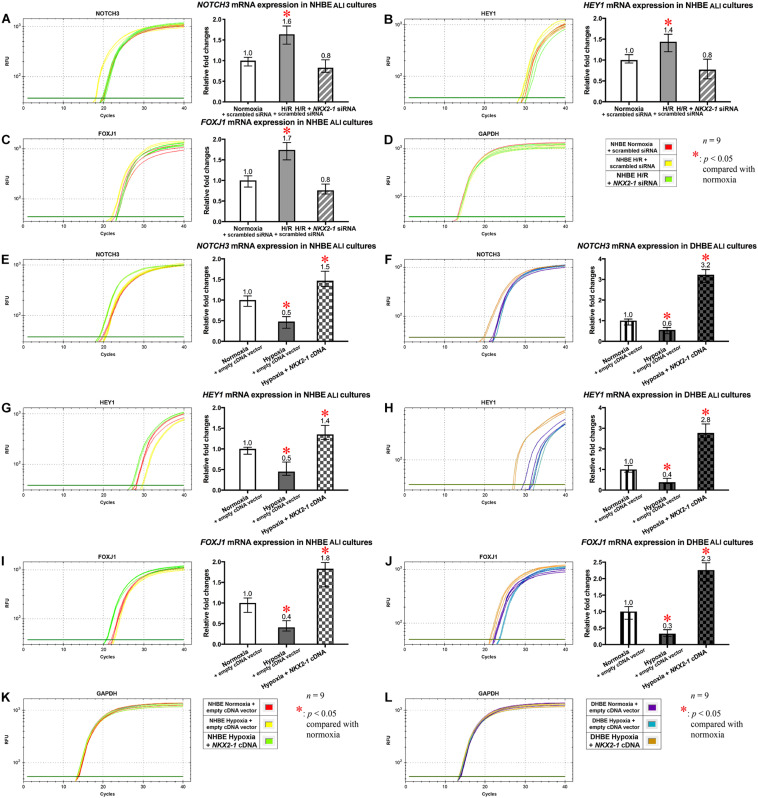
The mRNA levels of *NOTCH3*, *HEY1*, and *FOXJ1* were concordantly downregulated by *NKX2-1* siRNA transfection in the NHBE tissues cultured under intermittent H/R and concordantly upregulated by *NKX2-1* cDNA transfection in both the NHBE and DHBE tissues cultured under consecutive hypoxia. **(A–C)** Transfection of *NKX2-1* siRNA into the NHBE cells followed by ALI culturing under intermittent H/R consistently and significantly decreased the mRNA levels of *NOTCH3*, *HEY1*, and *FOXJ1* in the differentiated NHBE tissues compared to the levels in the NHBE tissues transfected with the scrambled negative control siRNA and cultured under intermittent H/R (which were indicated by asterisks) (*n* = 12, *p* < 0.05). Therefore, after *NKX2-1* siRNA transfection, the mRNA levels of *NOTCH3*, *HEY1*, and *FOXJ1* in the NHBE tissues cultured under intermittent H/R became comparable with the levels in the NHBE tissues cultured under normoxia (*n* = 12, *p* > 0.99). **(E–J)** Transfection of *NKX2-1* cDNA into both the NHBE and DHBE cells followed by ALI culturing under consecutive hypoxia consistently and significantly increased the mRNA levels of *NOTCH3*, *HEY1*, and *FOXJ1* more than 3-fold in the differentiated NHBE tissues **(E,G,I)** and more than 5-fold in the differentiated DHBE tissues **(F,H,J)** compared to the mRNA levels in the NHBE and DHBE tissues transfected with the scrambled negative control siRNA and cultured under consecutive hypoxia (*n* = 12, *p* < 0.001). Therefore, after *NKX2-1* cDNA transfection, the mRNA levels of *NOTCH3*, *HEY1* and *FOXJ1* increased more than 1.3-fold and more than 2.2-fold, respectively, in the NHBE and DHBE tissues cultured under consecutive hypoxia compared respectively to the levels in the NHBE and DHBE tissues cultured under normoxia (*n* = 12, *p* < 0.05 for the NHBE tissues and *p* < 0.001 for the DHBE tissues). The asterisks (^∗^) indicate *p* < 0.05 when comparing the HBE cells cultured under intermittent H/R or consecutive hypoxia with the same type of cells cultured under normoxia. **(D,K,L)** qPCR amplification curves of the mRNA levels of the reference gene GAPDH in NHBE tissues **(D,K)** and DHBE tissues **(L)**, respectively.

On the other hand, *NKX2-1* siRNA transfection into the NHBE cells followed by 18 days of ALI culture under intermittent H/R led to decreased expression of *NOTCH3*, *HEY1* and *FOXJ1* mRNAs to approximately the same levels in the NHBE tissues cultured under normoxia for 18 days (Figures 8A–C). For each group of the NHBE cells cultured under normoxia and H/R, three independent experiments of *NKX2-1* or scrambled siRNA transfection and qPCR analyses were performed, and *NOTCH3*, *HEY1* and *FOXJ1* mRNAs all exhibited consistent changes in the increases or decreases of the amplification cycle numbers among the three independent qPCR analyses when compared between different groups of HBE cells analyzed (Supplementary Figures S8A–C). In addition, after ALI culture under 9 days of consecutive hypoxia and 9 days of normoxia, *NKX2-1* cDNA transfection caused more than 1.4-fold increases in the expression levels of *NOTCH3*, *HEY1*, and *FOXJ1* mRNAs in both the NHBE and DHBE tissues compared to the HBE tissues cultured under normoxia for 18 days (Figures 8E–J). For each group of the NHBE and DHBE cells cultured under normoxia and 9-day consecutive hypoxia, three independent experiments of *NKX2-1* cDNA or empty cDNA vector transfection and qPCR analyses were performed, and *NOTCH3*, *HEY1*, and *FOXJ1* mRNAs all exhibited consistent changes in the increases or decreases of the amplification cycle numbers among the three independent qPCR analyses when compared between different groups of HBE cells analyzed (Supplementary Figures S8D–F). Our results indicated that, during ALI-induced differentiation of HBE cells, the expression levels of *NOTCH3*, *HEY1*, and *FOXJ1* mRNAs were co-upregulated by *NKX2-1* under both intermittent H/R and consecutive hypoxia.

### The Expression Levels of the Stem/Progenitor Cell Markers OCT4 and CC10 in Undifferentiated HBE Cells Are Respectively Co-regulated With the HIF1A/BMP4/NOTCH1/MKI67 and NKX2-1/NOTCH3/HEY1 Gene Modules Under Intermittent H/R or Consecutive Hypoxia

**FIGURE 9:**
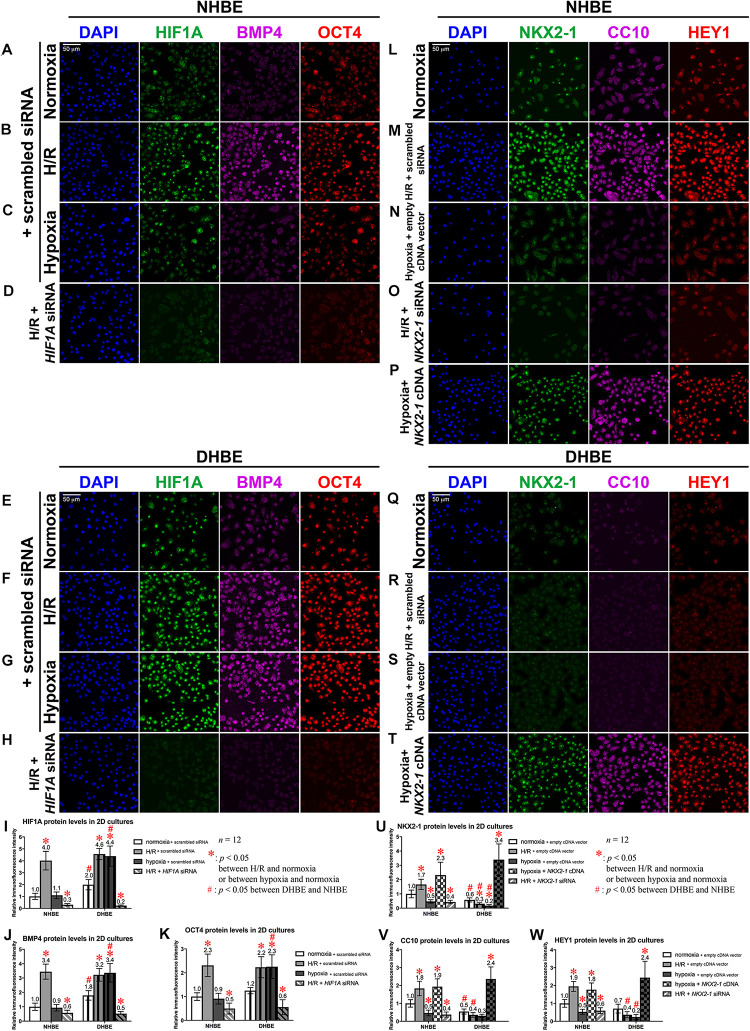
Colocalization and concordant regulation of the immunofluorescence signals of HIF1A, BMP4 and OCT4 proteins, and of the immunofluorescence signals of NKX2-1, CC10 and HEY1 proteins in both undifferentiated NHBE and DHBE cells. **(A–H)** Triple immunofluorescence staining for HIF1A (green), BMP4 (magenta), and OCT4 (red) in the submerged cultures of undifferentiated NHBE cells **(A–D)** and DHBE cells **(E–H)** revealed colocalization of HIF1A, BMP4 and OCT4 proteins in the same HBE cells. **(I–K)** Statistical charts showing concordant upregulation of the immunofluorescence intensities of HIF1A, BMP4 and OCT4 proteins by intermittent H/R in both the NHBE and DHBE cells. **(L–T)** Triple immunofluorescence staining for NKX2-1 (green), CC10 (magenta) and HEY1 (red) in the submerged cultures of undifferentiated NHBE cells **(L–P)** and DHBE cells **(Q–T)** revealed colocalization of NKX2-1, CC10 and HEY1 proteins in the same HBE cells. **(U–W)** Statistical charts showing concordant downregulation of the immunofluorescence intensities of NKX2-1, CC10, and HEY1 proteins by consecutive hypoxia in both the NHBE and DHBE cells. The scale bars in **(A,E,L,Q)** all represent 50 μm and respectively apply to panels **(A–H,L–T)**.

To further elucidate whether dysregulated expression of the ciliated and goblet cell-specific markers FOXJ1 and MUC5AC in the differentiated HBE cells was associated with aberrant expression of stem/progenitor cell markers, we analyzed protein and mRNA expression of the stem/progenitor cell markers OCT4 and CC10 in HBE cells in both the submerged and ALI cultures. Our immunostaining analyses revealed that, in the undifferentiated HBE cells in the submerged cultures, OCT4 was co-expressed with HIF1A and BMP4 in the same cells, and the changes in the immunofluorescence intensities of HIF1A, BMP4 and OCT4 were correlated with each other under intermittent H/R or consecutive hypoxia (Figures 9A–C,E–G, I–K), namely, while intermittent H/R significantly induced more than 2-fold increases in the protein levels of HIF1A, BMP4 and OCT4 in both the undifferentiated NHBE and DHBE cells, consecutive hypoxia significantly caused more than 1.8-fold increases in the levels of HIF1A, BMP4 and OCT4 in only the DHBE rather than NHBE cells (Figures 9I–K). On the other hand, CC10 was co-expressed with NKX2-1 and HEY1 in the same cells, and the changes in the immunofluorescence intensities of NKX2-1, CC10 and HEY1 were correlated with each other under intermittent H/R or consecutive hypoxia (Figures 9L–N,Q–S,U–W), namely, while consecutive hypoxia significantly decreased the protein levels of NKX2-1, CC10 and HEY1 by more than 40% in both the undifferentiated NHBE and DHBE cells, intermittent H/R significantly induced more than 1.6-fold increases in the levels of NKX2-1, CC10 and HEY1 in only the NHBE rather than DHBE cells (Figures 9U–W).

Our immunostaining analyses indicated that, transfection with *HIF1A* siRNA significantly decreased the immunofluorescence intensities of HIF1A, BMP4 and OCT4 by more than 75% in both the undifferentiated NHBE and DHBE cells cultured under intermittent H/R (Figures 9D,H,I–K), and transfection with *NKX2-1* cDNA significantly induced more than 3.3-fold increases in the protein levels of NKX2-1, CC10 and HEY1 in both the undifferentiated NHBE and DHBE cells cultured under consecutive hypoxia (Figures 9P,T,U–W), while transfection with *NKX2-1* siRNA significantly decreased the protein levels of NKX2-1, CC10 and HEY1 by more than 68% in the undifferentiated NHBE cells cultured under intermittent H/R (Figures 9O,U–W). The immunofluorescence analyses in the ALI-cultured and differentiated NHBE and DHBE tissues also revealed concordant upregulation of the protein levels of HIF1A and BMP4 in both the NHBE and DHBE tissues under intermittent H/R and in the DHBE tissues under consecutive hypoxia (Supplementary Figures S9A–C,E–G). Nonethless, the immunofluorescence intensities of OCT4-positive signals were dramatically reduced in the ALI-cultured HBE tissues compared to the HBE cells in the submerged cultures (Supplementary Figures S9A–C,E–G). Transfection with *HIF1A* siRNA was capable of significantly decreasing the immunofluorescence intensity of BMP4 in both the NHBE and DHBE tissues cultured under intermittent H/R (Supplementary Figures S9D,H). On the other hand, the protein levels of NKX2-1 and HEY1 were concordantly upregulated in the ALI-cultured NHBE tissues under intermittent H/R, and were concordantly downregulated in both the ALI-cultured NHBE and DHBE tissues under consecutive hypoxia (Supplementary Figures S9I–K,N–P). It is also noteworthy that the immunofluorescence intensities of CC10-positive signals were dramatically reduced in the ALI-cultured HBE tissues compared to the HBE cells in the submerged cultures (Supplementary Figures S9I–K,N–P). Transfection with NKX2-1 cDNA was capable of significantly increasing the immunofluorescence intensities of both HEY1 and CC10 in both the NHBE and DHBE tissues cultured under consecutive hypoxia (Supplementary Figures S9M,Q). Magnified immunofluorescence images with higher resolution further indicated colocalization of FOXJ1, NKX2-1 and HEY1 proteins in the nuclei of both NHBE and DHBE cells in the ALI cultures (Supplementary Figures S10A–I), and colocalization of MUC5AC, HIF1A and BMP4 proteins in both NHBE and DHBE cells in the ALI cultures (Supplementary Figures S10J–R). The decreases in the protein levels of FOXJ1, NKX2-1 and HEY1 were concordant with each other in both the NHBE and DHBE in the ALI cultures under 9-day consecutive hypoxia (Supplementary Figures S10C,H, S11C,D), and the increases in the protein levels of MUC5AC, HIF1A and BMP4 were concordant with each other in both the NHBE and DHBE in the ALI cultures under intermittent H/R (Supplementary Figures S10K,P, S11A,B). Transfection with *NKX2-1* cDNA was capable of significantly increased the immunofluorescence intensities of FOXJ1, NKX2-1, and HEY1 proteins in both the ALI-cultured NHBE and DHBE cells under consecutive hypoxia (Figures 8I,J and Supplementary Figures S10E,I, S11C,D), while transfection with *HIF1A* siRNA was capable of significantly decreased the immunofluorescence intensities of MUC5AC, HIF1A, and BMP4 proteins (Figures 7D,I and Supplementary Figures S10M,R, S11A,B).

**FIGURE 10:**
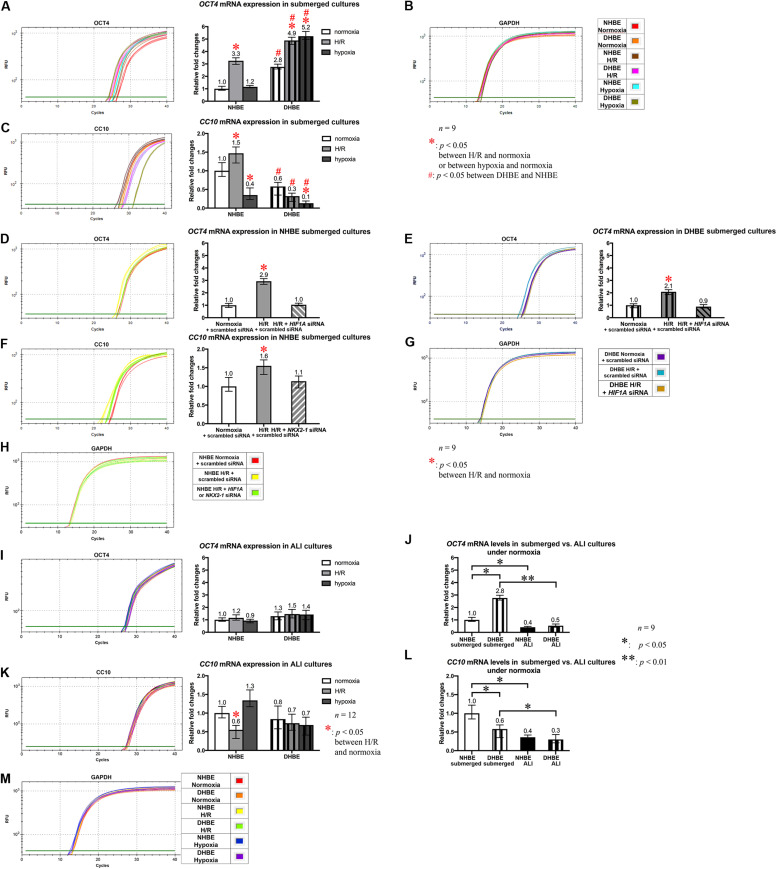
*OCT4* and *CC10* mRNA levels were respectively regulated by *HIF1A* and *NKX2-1* mRNA expression and were differentially regulated between the 2D and 3D cultures of HBE cells. **(A–C)** The mRNA levels of *OCT4* and *CC10* in the undifferentiated HBE cells were each concordantly regulated with the mRNA levels of *HIF1A*/*BMP4*/*NOTCH1*/*MKI67* and *NKX2-1*/*NOTCH3*/*HEY1* gene sets, respectively, in both undifferentiated and differentiated HBE cells (compare with Figure 4 and data not shown). **(D–F)** Transfection with *HIF1A* siRNA or *NKX2-1* siRNA each significantly decreased the intermittent H/R-induced *OCT4* or *CC10* expression in the undifferentiated NHBE cells to the level comparable to that under normoxia (*n* = 12, *p* > 0.99). **(I–L)** In the ALI cultures of HBE tissues, the mRNA levels of *OCT4* and *CC10* became comparable between the NHBE and DHBE tissues and between normoxia, intermittent H/R and consecutive hypoxia, except for the NHBE tissues cultured under intermittent H/R, in which *CC10* expression was significantly decreased compared to the other culturing conditions **(I,K)**. It is noteworthy that both *OCT4* and *CC10* mRNAs showed significantly decreased levels in the ALI cultures of HBE cells compared to the submerged cultures, and that in the submerged cultures, *OCT4* expression was significantly increased in the DHBE cells compared to the NHBE cells, in contrast to the significantly decreased expression of *CC10* in the DHBE cells **(J,L)**. In **(A–F,I,K)** the single asterisks (^∗^) indicate *p* < 0.05 when comparing the HBE cells cultured under intermittent H/R or consecutive hypoxia with the same type of cells cultured under normoxia, and the hashtags (#) indicate *p* < 0.05 when comparing the DHBE tissues with the NHBE tissues cultured under the same oxygen tension. In **(J,L)**, the single asterisks (^∗^) and double asterisk (^∗∗^) respectively indicate *p* < 0.05 and *p* < 0.01 when comparing between the NHBE and DHBE cells under the same culturing condition, or between the 2D and 3D cultures using the same type of HBE cells. **(B,G,H,M)** qPCR amplification curves of the mRNA levels of the reference gene *GAPDH* in the submerged cultures of both NHBE and DHBE cells **(B)** and of DHBE cells **(G)** and NHBE cells **(H)**, respectively, as well as in the ALI cultures of both NHBE and DHBE tissues **(M)**.

Our qPCR analyses also revealed that, in agreement with the changes in the immunostaining levels, the *OCT4* mRNA level was significantly increased by intermittent H/R in both the undifferentiated NHBE and DHBE cells and also significantly increased by consecutive hypoxia in the undifferentiated DHBE cells (Figure 10A), while the *CC10* mRNA level was significantly increased by intermittent H/R in the undifferentiated NHBE cells whereas significantly decreased by consecutive hypoxia in both the undifferentiated NHBE and DHBE cells (Figure 10C). In addition, transfection with *HIF1A* siRNA significantly decreased *OCT4* mRNA levels in both the undifferentiated NHBE and DHBE cells cultured under intermittent H/R to the same levels as cultured under normoxia (Figures 10D,E), and transfection with *NKX2-1* siRNA significantly decreased the *CC10* mRNA level in the undifferentiated NHBE cells cultured under intermittent H/R to the same level as cultured under normoxia (Figure 10F), suggesting that the expression levels of *OCT4* and *CC10* are subject to regulation by *HIF1A* and *NKX2-1* mRNA levels, respectively. However, in the ALI-cultured and differentiated NHBE and DHBE tissues, the *OCT4* mRNA expression levels were not significantly different between the normoxic, intermittent H/R and consecutive hypoxic culturing conditions (Figure 10I), and the mRNA levels of *OCT4* were significantly decreased in the ALI-cultured HBE tissues compared to the submerged and undifferentiated HBE cells (Figure 10J). On the other hand, *CC10* mRNA expression in the ALI cultures showed a significant reduction only in the differentiated NHBE tissues cultured under intermittent H/R, whereas showed neither significant difference between normoxia and consecutive hypoxia in the NHBE and DHBE tissues, nor between normoxia, intermittent H/R and consecutive hypoxia in the DHBE tissues (Figure 10K). Similar to *OCT4*, *CC10* mRNA also exhibited significantly reduced expression levels in the ALI-cultured HBE tissues in comparison with the submerged and undifferentiated HBE cells (Figure 10L). Quantitative PCR analyses revealed comparable mRNA expression levels of the reference housekeeping gene *GAPDH* between distinct groups of NHBE and DHBE cells cultured under normoxia, intermittent H/R or consecutive hypoxia, and transfected with either *HIF1A/NKX2-1* siRNA or scrambled siRNA (Figures 10B,G,H,M).

## Discussion

**FIGURE 11:**
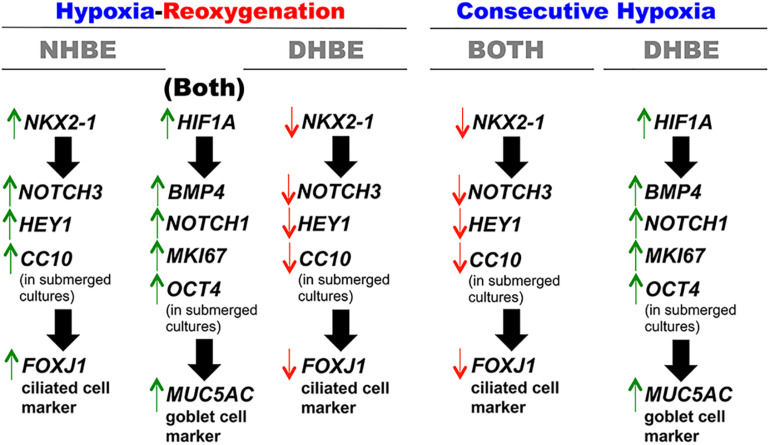
Differential regulation of the two distinct co-regulated gene modules in HBE cells cultured under intermittent H/R or consecutive hypoxia. Our study shows for the first time that the mRNA levels of the ciliated cell marker *FOXJ1* and goblet cell marker *MUC5A*C are regulated concordantly with the two distinct *NKX2-1*/*NOTCH3*/*HEY1*/*CC10* and *HIF1A*/*BMP4*/*NOTCH1*/*MKI67*/*OCT4* co-regulated gene modules, respectively, in HBE cells cultured under different oxygen tensions *in vitro*. As for common co-regulation in different types of HBE cells, the levels of *HIF1A*, *BMP4*, *NOTCH1*, *MKI67*, *OCT4* and *MUC5AC* mRNAs are all upregulated by intermittent 24/24-h cycles of H/R and the levels of *NKX2-1*, *NOTCH3*, *HEY1*, *CC10* and *FOXJ1* mRNAs are all downregulated by 9-day consecutive hypoxia in both NHBE and DHBE cells. As for differential regulation in different types of HBE cells, the levels of *NKX2-1*, *NOTCH3*, *HEY1*, *CC10*, and *FOXJ1* mRNAs are co-upregulated in NHBE cells and co-downregulated in DHBE cells, while the levels of *HIF1A*, *BMP4*, *NOTCH1*, *MKI67*, *OCT4*, and *MUC5AC* mRNAs are co-upregulated in DHBE cells only.

According to the results of our immunostaining and qPCR analyses, *NKX2-1* and *HIF1A* respectively regulate the expression of the two distinct *NOTCH3/HEY1/CC10/FOXJ1* and *BMP4/NOTCH1/MKI67/OCT4/MUC5AC* gene modules in the HBE cells cultured *in vitro* under consecutive hypoxia or intermittent H/R. The genes which are respectively co-upregulated and co-downregulated by intermittent H/R and consecutive hypoxia in the NHBE and DHBE cells are summarized in Figure 11. We found that there was no significant difference in the exacerbating effects on the mucociliary differentiation defects of DHBE cells between intermittent H/R and consecutive hypoxia, as both intermittent H/R and consecutive hypoxia significantly increased expression of the goblet cell marker MUC5AC and significantly decreased expression of the ciliated cell marker FOXJ1. Our findings are in agreement with the previous studies showing significantly increased hypoxic and apoptotic regions in the lung tissue sections in a mouse model of COPD manifestations (Weng et al., 2013). In addition, a recent study has also reported both significantly decreased percentages of β-tubulin IV^+^ ciliated cells and significantly increased percentages of MUC5AC^+^ goblet cells in the large airway epithelium from COPD patients (Gohy et al., 2019). On the other hand, intermittent H/R and consecutive hypoxia exert apparently different effects on the mucociliary differentiation of NHBE cells, as intermittent H/R promoted expression of both the ciliated cell marker FOXJ1 and the goblet cell marker MUC5AC, whereas consecutive hypoxia significantly suppressed ciliated cell differentiation without affecting goblet cell differentiation.

Although NKX2-1 and NOTCH3 are both well known as important markers and regulators of lung development, differentiation and homeostasis (Minoo et al., 1997; Snyder et al., 2013; Mori et al., 2015; Herriges et al., 2017), NKX2-1 has been shown to upregulate NOTCH3 signaling and *HEY1* expression in T and B lymphocytes but not in the airway epithelium (Nagel et al., 2009; Nagel et al., 2013). Interestingly, decreased mRNA expression of both *Foxj1* and *Nkx2-1* was observed in the lung tissues derived from the offspring of cigarette smoke-exposed maternal mice (Song, 2016). Decreased *NKX2-1* expression has also been observed in human lung tissues derived from COPD patients and is associated with cigarette smoking, WNT-5A overexpression, and distal-to-proximal airway re-patterning (Baarsma et al., 2017; Yang et al., 2017; Huang et al., 2019). It is noteworthy that transcription of the Clara cell marker gene *CC10* (also known as *CCSP* or *CC16*) has been reported to be directly and synergistically activated by binding of NKX2-1, C/EBPα and FOXA (HNF-3) transcription factors on the proximal promoter of *CC10* (Cassel et al., 2002; Ramsay et al., 2003; Minoo et al., 2007). In association with decreased *NKX2-1* expression in the COPD airways, reduced Clara (club) cell numbers and CC10 expression/secretion have been observed in both the peripheral and central airways of COPD patients and in cigarette smoke-exposed mouse and monkey airways (Shijubo et al., 1997; Pilette et al., 2001; Chen J. et al., 2007; Polverino et al., 2013; Barnes, 2015; Zhu et al., 2015). In comparison with NHBE cells, our immunostaining and qPCR analyses also revealed significantly decreased levels of NKX2-1 protein and mRNA in both the submerged and ALI-cultured DHBE cells (Figures 5F, 9U, and data not shown), as well as significantly decreased levels of CC10 protein and mRNA in the submerged DHBE cells (Figures 9V, 10C, and data not shown). In addition, in the submerged cultures, NKX2-1 and CC10 were co-upregulated by intermittent H/R and co-downregulated by consecutive hypoxia in NHBE cells, whereas co-downregulated by both intermittent H/R and consecutive hypoxia in DHBE cells (Figures 9U,V, 10C, and data not shown).

In contrast to the co-regulation between NKX2-1 and CC10 in the submerged cultures, the changes in the mRNA levels of *NKX2-1* and *CC10* under intermittent H/R or consecutive hypoxia were no longer concordant with each other in the ALI cultures of HBE cells (compare Figure 5F with Figure 10K). Interestingly, there were nearly equivalent levels of *CC10* mRNA between NHBE and DHBE tissues and between normoxia, intermittent H/R and consecutive hypoxia in the ALI cultures, except for the NHBE tissues cultured under intermittent H/R, which showed a more than 44% reduction in the *CC10* mRNA level compared to the NHBE tissues cultured under normoxia (Figure 10K). Given that CC10^+^ Clara (club) cells have been demonstrated to be capable of differentiating into both goblet and ciliated cells (Chen et al., 2009; Rawlins et al., 2009; Tompkins et al., 2009; Reynolds and Malkinson, 2010; Chu et al., 2019), it is plausible that significantly decreased *CC10* mRNA levels in the ALI-cultured HBE tissues compared to the submerged and undifferentiated HBE cells (Figure 10L) are attributable to increased differentiation of *CC10*-expressing cells. While the MUC5AC protein and mRNA levels in the DHBE tissues cultured under intermittent H/R and consecutive hypoxia showed nearly 2-fold increases compared to the DHBE tissues cultured under normoxia (Figures 2F, 5E), the FOXJ1 protein and mRNA levels in the DHBE tissues cultured under intermittent H/R and consecutive hypoxia showed approximately 50% decreases compared to the DHBE tissues cultured under normoxia (Figures 2E, 5I), hence it is reasonable that the *CC10* mRNA levels were comparable between the DHBE tissues cultured under normoxia, intermittent H/R and consecutive hypoxia (Figure 10K). On the other hand, the significantly increased expression of both FOXJ1 and MUC5AC in the NHBE tissues cultured under intermittent H/R compared to the NHBE tissues cultured under normoxia or consecutive hypoxia (Figures 2E,F, 5E,I) suggested increased differentiation into both goblet and ciliated cells at the expense of a decreased number of CC10^+^ progenitor cells, and thus the *CC10* mRNA level was significantly decreased in the NHBE tissues cultured under intermittent H/R compared to the other culturing conditions (Figure 10K). However, in the NHBE tissues cultured under consecutive hypoxia compared to those cultured under normoxia, there were comparable levels of *CC10* mRNA and MUC5AC mRNA/protein expression (Figures 2F, 5E, 10K), in spite of the presence of more than 50% reductions in the FOXJ1 mRNA and protein levels (Figures 2E, 5I). Taken together, *CC10* expression in the ALI-cultured and differentiated HBE tissues is influenced by both *FOXJ1* and *MUC5AC* expression.

Although both NOTCH1 and NOTCH3 have been implicated in regulating the balanced differentiation of airway basal cells between the secretory and ciliated cell fates (Tsao et al., 2009; Gomi et al., 2015), both NOTCH3 and HEY1 mRNA expression was significantly decreased in the small airway epithelium of COPD patients (Tilley et al., 2009), while increased expression levels of activated NOTCH1 and the effector HEY2 were observed in COPD airways and mutant murine lungs and were associated with decreased numbers of ciliated cells and increased goblet cell metaplasia and mucus overproduction (Guseh et al., 2009; Boucherat et al., 2012), suggesting that NOTCH1 and NOTCH3 may play distinct roles in regulating airway epithelial differentiation and proliferation. Previous studies have demonstrated that enhanced squamous metaplasia and goblet cell hyperplasia as well as reduced ciliogenesis are all correlated with increased NOTCH signaling and are implicated in the COPD airways (Pilette et al., 2001; Guseh et al., 2009; Boucherat et al., 2012; Barnes, 2015; Gohy et al., 2019; Belgacemi et al., 2020). It has also been shown recently that reduced ciliogenesis in COPD airways is associated with overexpressed TGF-β1 and deficient Sonic hedgehog (Shh)/Gli2/Smo signaling (Gohy et al., 2019; Belgacemi et al., 2020). In this study, we show for the first time that *NOTCH1* and *NOTCH3* expression levels in the COPD airway epithelium are differentially regulated and respectively correlated with overexpression of the goblet cell marker *MUC5AC* and deficiency of the ciliated cell marker *FOXJ1* (Figures 5, 7, 8).

On the other hand, HIF1α has been reported to directly upregulate BMP4 expression in various types of cells and indirectly stabilize NOTCH1 protein and activate NOTCH1-HES1 signaling in human glioma stem-like cells and T lymphocytes (Wu and Paulson, 2010; Zou et al., 2013; Wang et al., 2015; Pramono et al., 2016; Man et al., 2018). In A549 lung adenocarcinoma cells exposed to cadmium, HIF1α has been shown to upregulate the expression level of the intracellular domain (ICD) but not the transmembrane subunit (NTM) of NOTCH1, and the regulation is independent of the HIF1α transcriptional activity (Fujiki et al., 2017). It is likely that HIF1α stabilizes NOTCH1 ICD and potentiates NOTCH1 signaling via direct protein-protein interactions, as demonstrated in another previous study (Gustafsson et al., 2005). Furthermore, BMP and NOTCH1 have been corroborated to synergistically activate multiple gene transcription in various tissues, including transactivation of the *Hey1*, *Hes1*, *Hes5*, *Fli1*, and *Sox2* promoters in myoblasts, fibroblasts, cerebellar primordium, *Xenopus* and zebrafish embryos, as well as endothelial cells, respectively (Dahlqvist et al., 2003; Masserdotti et al., 2010; Mouillesseaux et al., 2016; Wu et al., 2019). In addition to the crosstalk between BMP and NOTCH signaling, BMP6 and BMP9 were also shown to induce expression of Notch1 protein and its ligands Jagged1 and 2 in cerebral endothelial cells (Wu et al., 2019). In our study, we found that increased *HIF1A* expression was indispensable for upregulation of both *BMP4* and *NOTCH1* mRNA expression in the differentiated HBE tissues under intermittent H/R (Figures 7A,B,F,G), whereas it remains to be studied whether *BMP4* alone was capable of upregulating *NOTCH1* expression in the HBE cells.

While HIF1α has been shown to promote proliferation of goblet cells and alveolar type II (AT2) cells in the airway epithelium as well as pulmonary artery smooth muscle cells (PASMCs) and various types of lung tumor cells (Polosukhin et al., 2011; Hubbi and Semenza, 2015; McClendon et al., 2017; de La Garza et al., 2018; Dabral et al., 2019; Raoof and Daoud, 2019), BMP4 has been reported to inhibit proliferation of both human and mouse airway basal progenitor cells, mouse alveolar AT2 cells and tracheal epithelial cells, as well as the immortalized human airway epithelial cell line BEAS-2B (Hyatt et al., 2002; Molloy et al., 2008; Tadokoro et al., 2016; Chung et al., 2018; Zuo et al., 2019), although BMP4 also plays the same role as HIF1α in promoting PASMC proliferation in response to hypoxia (Frank et al., 2005). In this study, however, we found that the changes in the mRNA levels of both *HIF1A* and *BMP4* were correlated with the changes in the mRNA levels of the proliferation marker *MKI67* under intermittent H/R and consecutive hypoxia in the ALI cultures of both differentiated NHBE and DHBE tissues (Figures 5A,B,D). Besides, *HIF1A* knockdown significantly decreased both BMP4 and *MKI67* expression in both the differentiated and undifferentiated NHBE and DHBE cells under intermittent H/R (Figures 7A,C,F,H, 9J, and data not shown), indicating that BMP4 expression and cell proliferation are co-regulated by the *HIF1A* mRNA level in the ALI-cultured and differentiated HBE cells.

In addition to regulation of cell proliferation, HIF1α has been shown to bind directly to the hypoxia-response elements within the MUC5AC promoter in both human bronchial and nasal epithelial cells, and increased co-expression of HIF1α and MUC5AC has been detected in both the regions of goblet cell hyperplasia in the COPD airway epithelium and the sinus mucosa from chronic sinusitis patients (Polosukhin et al., 2011; Zhou et al., 2012; Kim et al., 2014), while BMP4 has been reported to induce *MUC5AC* mRNA expression in the lung epithelium-differentiated mouse embryonic stem cells and activate MUC5AC protein expression in the immortalized human esophageal squamous epithelial cell line HET-1A via KLF4 upregulation (Ninomiya et al., 2013; Yan et al., 2016). In the differentiated NHBE and DHBE tissues cultured under intermittent H/R and consecutive hypoxia *in vitro*, our results indicated that the changes in the mRNA levels of both *HIF1A* and *BMP4* were correlated with the changes in both the mRNA and protein levels of MUC5AC (Figures 2F, 5A,B,E), in agreement with the previous studies that both HIF1α and BMP4 upregulate MUC5AC expression in various cell types (Polosukhin et al., 2011; Zhou et al., 2012; Ninomiya et al., 2013; Kim et al., 2014; Yan et al., 2016).

It is noteworthy that HIF1α and BMP4 have both been reported not only to promote MUC5AC expression, but also to upregulate expression of OCT4 (Qi et al., 2004; Zhang K. et al., 2010; Chen et al., 2011; Mathieu et al., 2011; Iida et al., 2012; Li et al., 2016; Liu et al., 2020), which is a pluripotency gene and a marker for lung and cancer stem cells (Ling et al., 2006; Chen Y. et al., 2007; Chen et al., 2008; Gonzalez et al., 2009; Karoubi et al., 2009; Chiou et al., 2010; Zhang X. et al., 2010; Khatri et al., 2012; Bora-Singhal et al., 2015; Jen et al., 2017). In fact, our immunostaining and qPCR analyses revealed that, in the submerged cultures, the significant increases in both the protein and mRNA levels of OCT4 by intermittent H/R in both the NHBE and DHBE cells and by consecutive hypoxia in the DHBE cells were concordant with the significant increases in both the protein and mRNA levels of HIF1A and BMP4 (Figures 9I−K, 10A, and data not shown). In addition, *HIF1A* knockdown in both the submerged NHBE and DHBE cell cultures under intermittent H/R significantly decreased both the protein and mRNA levels of OCT4 (Figures 9K, 10D,E), indicating that *HIF1A* mRNA is indispensable for OCT4 expression in the undifferentiated HBE cells. In the submerged cultures under normoxia, although the *OCT4* mRNA level showed a nearly 3-fold increase in DHBE cells compared to the level in NHBE cells (Figure 10A), the OCT4 protein level was comparable between NHBE and DHBE cells (Figure 9K). Given that multiple lines of evidence have shown the positive correlation between COPD and lung cancers (Raviv et al., 2011; Durham and Adcock, 2015; Spyratos et al., 2017; Young and Hopkins, 2018; Parris et al., 2019), which are generally characterized by increased OCT4 protein expression in the malignant airway epithelial tissues (Chen et al., 2008; Karoubi et al., 2009; Chiou et al., 2010; Zhang X. et al., 2010; Bora-Singhal et al., 2015), it is plausible that increased *OCT4* mRNA expression in the undifferentiated DHBE cells, which preceded increased OCT4 protein expression, may be a predisposing factor for lung cancer development in the COPD population. On the other hand, surprisingly, the *OCT4* mRNA levels were comparable among the differentiated NHBE and DHBE tissues cultured under normoxia, intermittent H/R or consecutive hypoxia (Figure 10I), which may be primarily attributable to the dramatically reduced expression levels of *OCT4* in the ALI-cultured and differentiated HBE tissues, which showed at least 50% decreases compared to the levels in the submerged and undifferentiated HBE cells (Figure 10J). It remains to be studied whether *OCT4* mRNA expression is generally suppressed during the process of airway epithelial differentiation, or *OCT4* transcription is specifically downregulated by some special chemical component(s) or protein factor(s) within the differentiation medium used in this study.

Taken together, our study shows for the first time that consecutive hypoxia decreased expression of the signaling factors *NOTCH3* and *HEY1*, the secretoglobulin gene *CC10* and the ciliogenesis-inducing transcription factor gene *FOXJ1* via *NKX2-1* mRNA downregulation, while intermittent H/R increased expression of the signaling factors *BMP4* and *NOTCH1*, the cell proliferation antigen gene *MKI67*, the stem cell marker gene *OCT4* and the predominant airway mucin gene *MUC5AC* via *HIF1A* mRNA upregulation. Our findings suggest that the putatively long-term (i.e., over 48 h) situation of ischemia-reperfusion concomitant with lung transplantation (Laubach and Sharma, 2016), which is usually inevitable for end-staged COPD patients (Aziz et al., 2010), may in fact impose even more exacerbating effects on the damaged COPD airway epithelium by further increasing mucus production and suppressing generation of ciliated cells. On the other hand, our results also suggest clinical application of HIF1A inhibitors, such as those reported by previous studies including EZN-2968 and CAY10585, which specifically and respectively inhibit *HIF1A* mRNA expression and protein synthesis/activity without affecting HIF2 (Yu et al., 2017), as potential drugs for inhibiting mucus overproduction in the COPD airways, as well as the application of *NKX2-1* gene delivery via viral vector transduction or nanoparticle delivery for activating the capacity of generating more ciliated cells in the COPD bronchial epithelium. Future studies will be required to further decipher the mutual regulatory mechanisms between *NOTCH3*, *HEY1*, *CC10*, and *FOXJ1*, and between *BMP4*, *NOTCH1*, *MKI67*, *OCT4*, and *MUC5AC*, respectively, during the distinct phases of proliferation and differentiation in both healthy and diseased human airway epithelial cells.

## Data Availability Statement

All datasets generated for this study are included in the article/Supplementary Material.

## Author Contributions

Y-YY performed most of the replicates of all experimental analyses and all statistical analyses in this study, as well as made requested revisions on the manuscript. C-JL and C-CW performed each experiment for 1–2 times, and contributed to part of the data analyses. C-MC provided the technical and material support. W-JK contributed to the technical support. Y-HC conceived and designed the study, obtained the funding, supervised all the experiments, contributed to the data analyses, and wrote the manuscript. All authors contributed to the article and approved the submitted version.

## Conflict of Interest

The authors declare that the research was conducted in the absence of any commercial or financial relationships that could be construed as a potential conflict of interest.

## Supplementary Material

FIGURE S1 Statistical dot plots of the relative protein and mRNA levels of FOXJ1 and MUC5AC in different groups of NHBE and DHBE cells in the ALI and submerged cultures. **(A,B)** Comparison of the percentages of FOXJ1^+^ and MUC5AC^+^ cells in the immunostaining analyses in the ALI and submerged cultures between three different groups of NHBE cells (NHBE1, NHBE2, and NHBE3) and three different groups of DHBE cells (DHBE1, DHBE2, and DHBE3). **(C,D)** Comparison of the mRNA levels of FOXJ1 and MUC5AC in the ALI and submerged cultures between three different groups of NHBE cells (NHBE1, NHBE2, and NHBE3) and three different groups of DHBE cells (DHBE1, DHBE2 and DHBE3). The singlet asterisk (^∗^) indicates *p* < 0.05 as compared between the two different groups of cells within the same type (i.e., NHBE2 vs. NHBE3 or DHBE1 vs. DHBE2), while the triplet asterisk (^∗∗∗^) indicates *p* < 0.001 between the submerged cultures and ALI cultures. The hashtag (#) indicates *p* < 0.05 when comparing the DHBE tissues with the NHBE tissues cultured under the same oxygen tension, and the ampersand (&) indicates *p* < 0.05 when compared to the same type of cells cultured under normoxia.

FIGURE S2 Real-time qPCR amplification curves of *HIF1A*, *BMP4*, *NOTCH1*, *MKI67*, and *MUC5AC* mRNAs in the ALI-cultured NHBE and DHBE cells for the second and third independent experiments.

FIGURE S3 Real-time qPCR amplification curves of *NKX2-1*, *NOTCH3*, *HEY1* and *FOXJ1* mRNAs in the ALI-cultured NHBE and DHBE cells for the second and third independent experiments.

FIGURE S4 Real-time qPCR amplification curves of *HIF1A* and *NKX2-1* mRNAs in the ALI-cultured NHBE and DHBE. **(A)** Comparison of the HIF1A mRNA levels with scrambled siRNA or *HIF1A* siRNA transfection. **(B)** Comparison of the HIF1A mRNA levels with scrambled siRNA or *NKX2-1* siRNA transfection. **(C)** Comparison of the HIF1A mRNA levels with scrambled siRNA or *NKX2-1* cDNA transfection.

FIGURE S5 Statistical dot plots showing *HIF1A* and *NKX2-1* mRNA levels in the ALI-cultured NHBE and DHBE cells transfected with *HIF1A* siRNA, *NKX2-1* siRNA or *NKX2-1* cDNA. The singlet asterisk (^∗^) indicates *p* < 0.05 and the doublet asterisk (^∗∗^) indicates *p* < 0.01 as compared between the two different groups of cells within the same type (i.e., NHBE2 vs. NHBE3 or DHBE1 vs. DHBE2). The hashtag (#) indicates *p* < 0.05 when comparing the DHBE tissues with the NHBE tissues cultured under the same oxygen tension, and the ampersand (&) indicates *p* < 0.05 when compared to the same type of cells cultured under normoxia.

FIGURE S6 Statistical dot plots showing the percentages of FOXJ1 + and MUC5AC + NHBE and DHBE cells in the ALI cultures under intermittent H/R or consecutive hypoxia. The singlet asterisk (^∗^) indicates *p* < 0.05 and the doublet asterisk (^∗∗^) indicates *p* < 0.01 as compared between the two different groups of cells within the same type (i.e., NHBE2 vs. NHBE3 or DHBE1 vs. DHBE2). The hashtag (#) indicates *p* < 0.05 when comparing the DHBE tissues with the NHBE tissues cultured under the same oxygen tension, and the ampersand (&) indicates *p* < 0.05 when compared to the same type of cells cultured under normoxia.

FIGURE S7 Real-time qPCR amplification curves of *BMP4*, *NOTCH1*, *MKI67*, and *MUC5AC* mRNAs in the ALI-cultured NHBE and DHBE cells transfected with *HIF1A* or scrambled siRNA for the second and third independent experiments.

FIGURE S8 Real-time qPCR amplification curves of *NOTCH3*, *HEY1*, and *FOXJ1* mRNAs in the ALI-cultured NHBE and DHBE cells transfected with *NKX2-1* siRNA or *NKX2-1* cDNA for the second and third independent experiments.

FIGURE S9 Colocalization and concordant regulation of the immunofluorescence signals of HIF1A, BMP4 and OCT4 proteins, and of the immunofluorescence signals of NKX2-1, CC10 and HEY1 proteins in both ALI-cultured NHBE and DHBE cells. **(A−H)** Triple immunofluorescence staining for HIF1A (green), BMP4 (magenta) and OCT4 (red) in the ALI cultures of differentiated NHBE cells **(A−D)** and DHBE cells **(E−H)** revealed colocalization of HIF1A, BMP4 and OCT4 proteins in the same HBE cells. **(I−Q)** Triple immunofluorescence staining for NKX2-1 (green), CC10 (magenta) and HEY1 (red) in the ALI cultures of differentiated NHBE cells **(I−M)** and DHBE cells **(N−Q)** revealed colocalization of NKX2-1, CC10 and HEY1 proteins in the same HBE cells. The scale bars in **(A,E,I,N)** all represent 200 μm and respectively apply to **(A–Q)**.

FIGURE S10 Colocalization of the immunofluorescence signals of FOXJ1, NKX2-1 and HEY1 proteins, and of the immunofluorescence signals of MUC5AC, HIF1A and BMP4 proteins in both ALI-cultured NHBE and DHBE cells. **(A−I)** Triple immunofluorescence staining for FOXJ1 (green), NKX2-1 (magenta) and HEY1 (red) in the ALI cultures of differentiated NHBE cells **(A−E)** and DHBE cells **(F−I)** revealed colocalization of FOXJ1, NKX2-1 and HEY1 proteins in the same HBE nuclei. **(J−R)** Triple immunofluorescence staining for MUC5AC (magenta), HIF1A (green) and BMP4 (red) in the ALI cultures of differentiated NHBE cells **(J−M)** and DHBE cells **(O−R)** revealed colocalization of MUC5AC, HIF1A, and BMP4 proteins in the same HBE cells. The scale bars in **(A,F,J,O)** all represent 5 μm and respectively apply to **(A–R)**.

FIGURE S11 Statistical dot plots of the relative protein levels of HIF1A, BMP4, NKX2-1 and HEY1 in different groups of NHBE and DHBE cells in the ALI cultures. Comparison of the relative immunofluorescence intensities of HIF1A **(A)**, BMP4 **(B)**, NKX2-1 **(C)**, and HEY1 **(D)** in the immunostaining analyses in the ALI cultures between three different groups of NHBE cells (NHBE1, NHBE2 and NHBE3) and three different groups of DHBE cells (DHBE1, DHBE2 and DHBE3). The singlet asterisk (*) indicates *p* < 0.05, the doublet asterisk (**) indicates *p* < 0.01, and the triplet asterisk (***) indicates *p* < 0.001 as compared between the two different groups of cells within the same type (i.e., NHBE2 vs. NHBE3 or DHBE1 vs. DHBE2). The hashtag (#) indicates *p* < 0.05 when comparing the DHBE tissues with the NHBE tissues cultured under the same oxygen tension, and the ampersand (&) indicates *p* < 0.05 when compared to the same type of cells cultured under normoxia.

FIGURE S12 Statistical dot plots of the relative protein levels of OCT4 and CC10 in different groups of NHBE and DHBE cells in the ALI and submerged cultures. **(A,B)** Comparison of the relative immunofluorescence intensities of OCT4 in the submerged **(A)** and ALI **(B)** cultures between three different groups of NHBE cells (NHBE1, NHBE2 and NHBE3) and three different groups of DHBE cells (DHBE1, DHBE2 and DHBE3). **(C,D)** Comparison of the relative immunofluorescence intensities of CC10 in the submerged **(C)** and ALI **(D)** cultures between three different groups of NHBE cells (NHBE1, NHBE2 and NHBE3) and three different groups of DHBE cells (DHBE1, DHBE2 and DHBE3). The singlet asterisk (*) indicates *p* < 0.05, the doublet asterisk (**) indicates *p* < 0.01, and the triplet asterisk (***) indicates *p* < 0.001 as compared between the two different groups of cells within the same type (i.e., NHBE2 vs. NHBE3 or DHBE1 vs. DHBE2). The hashtag (#) indicates *p* < 0.05 when comparing the DHBE tissues with the NHBE tissues cultured under the same oxygen tension, and the ampersand (&) indicates *p* < 0.05 when compared to the same type of cells cultured under normoxia.
