# Supplementary figures and images for: Consecutive Hypoxia Decreases Expression of NOTCH3, HEY1, CC10, and FOXJ1 via NKX2-1 Downregulation and Intermittent Hypoxia-Reoxygenation Increases Expression of BMP4, NOTCH1, MKI67, OCT4, and MUC5AC via HIF1A Upregulation in Human Bronchial Epithelial Cells
Source: Front Cell Dev Biol. 2020 Sep 4;8:572276. doi: 10.3389/fcell.2020.572276 (PMC7500169; doi:10.3389/fcell.2020.572276)

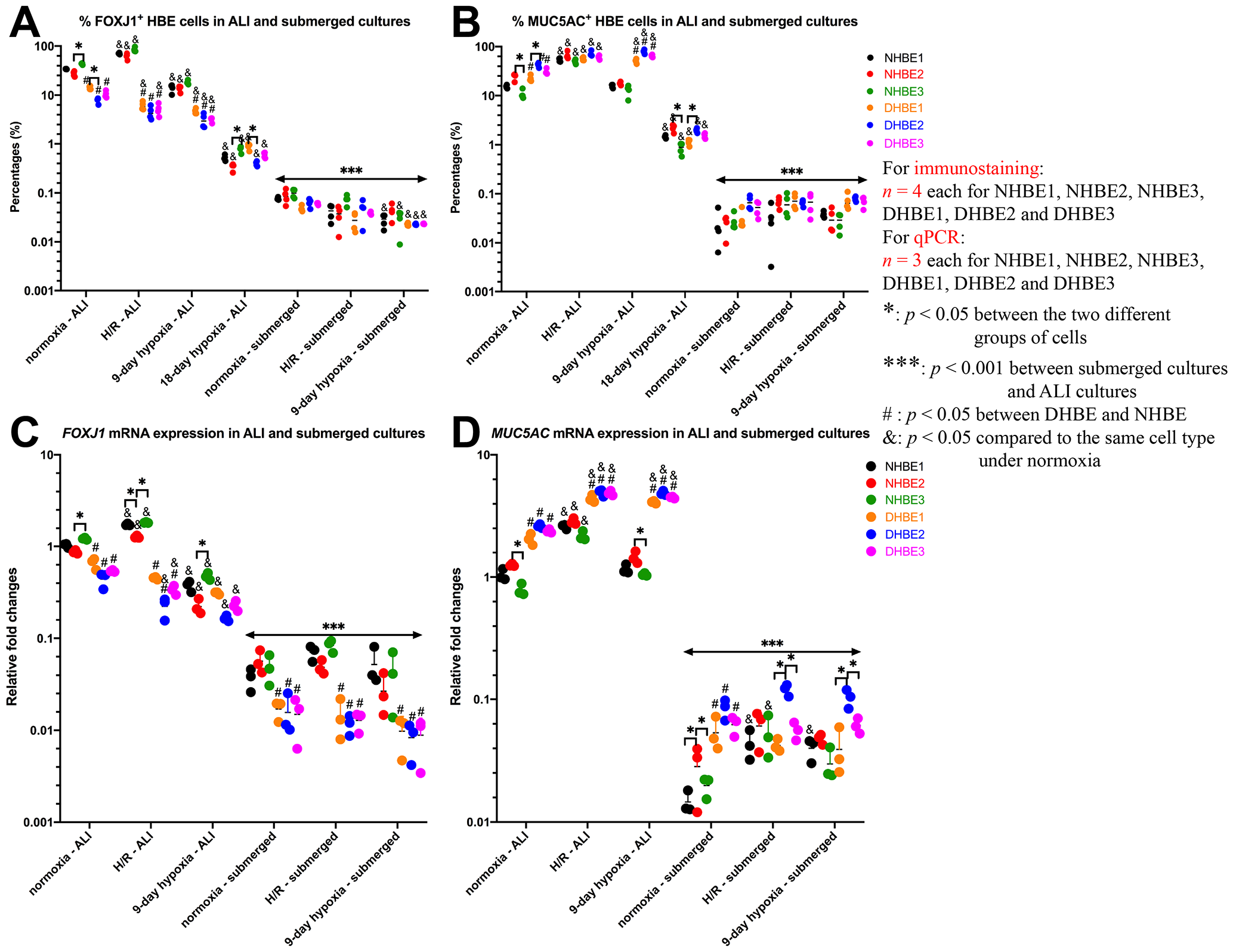

Supplement: FIGURE S1 — Statistical dot plots of the relative protein and mRNA levels of FOXJ1 and MUC5AC in different groups of NHBE and DHBE cells in the ALI and submerged cultures. (A,B) Comparison of the percentages of FOXJ1+ and MUC5AC+ cells in the immunostaining analyses in the ALI and submerged cultures between three different groups of NHBE cells (NHBE1, NHBE2, and NHBE3) and three different groups of DHBE cells (DHBE1, DHBE2, and DHBE3). (C,D) Comparison of the mRNA levels of FOXJ1 and MUC5AC in the ALI and submerged cultures between three different groups of NHBE cells (NHBE1, NHBE2, and NHBE3) and three different groups of DHBE cells (DHBE1, DHBE2 and DHBE3). The singlet asterisk (∗) indicates p < 0.05 as compared between the two different groups of cells within the same type (i.e., NHBE2 vs. NHBE3 or DHBE1 vs. DHBE2), while the triplet asterisk (∗∗∗) indicates p < 0.001 between the submerged cultures and ALI cultures. The hashtag (#) indicates p < 0.05 when comparing the DHBE tissues with the NHBE tissues cultured under the same oxygen tension, and the ampersand (&) indicates p < 0.05 when compared to the same type of cells cultured under normoxia. [file Image_1.TIF]

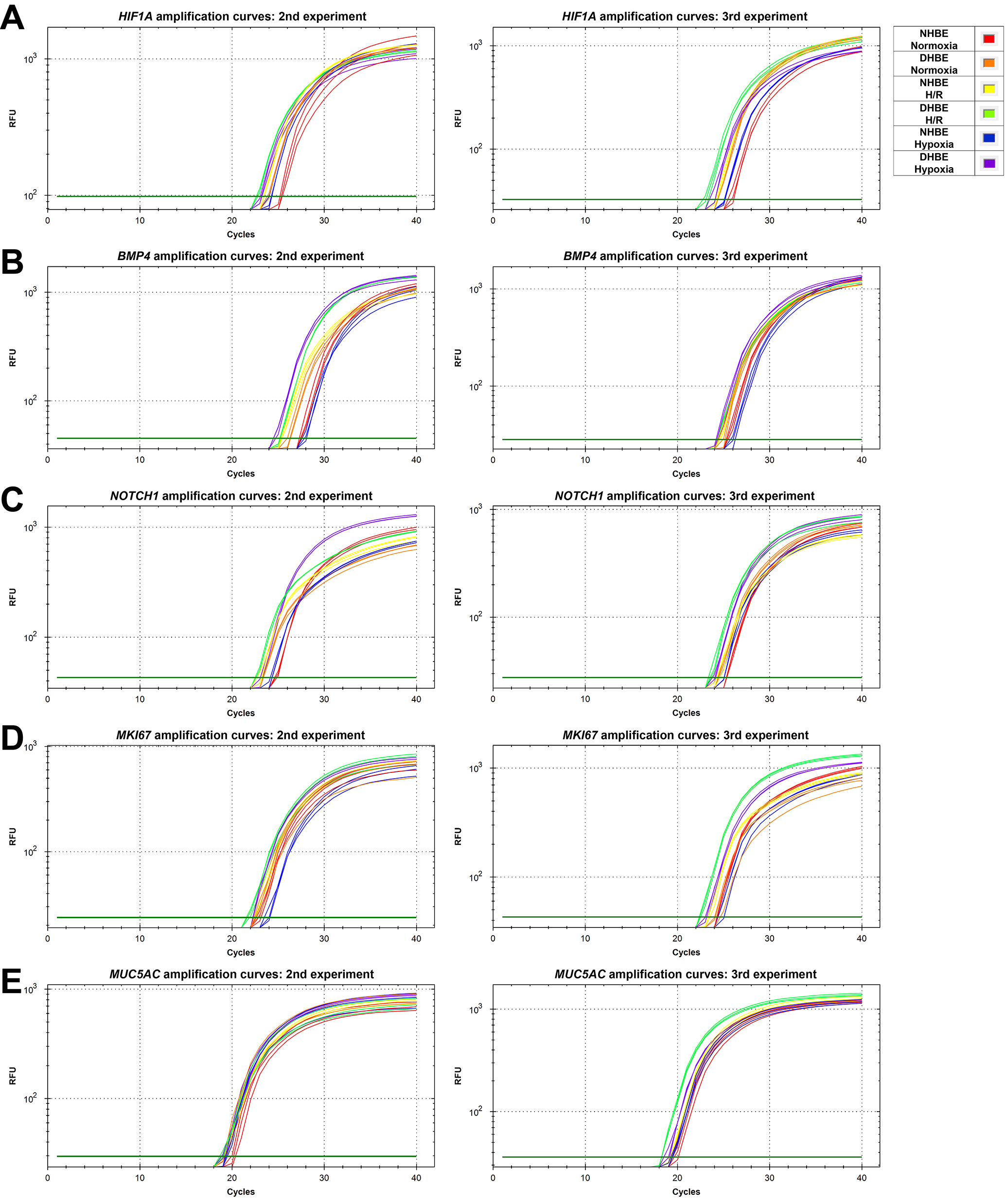

Supplement: FIGURE S2 — Real-time qPCR amplification curves of HIF1A, BMP4, NOTCH1, MKI67, and MUC5AC mRNAs in the ALI-cultured NHBE and DHBE cells for the second and third independent experiments. [file Image_2.TIF]

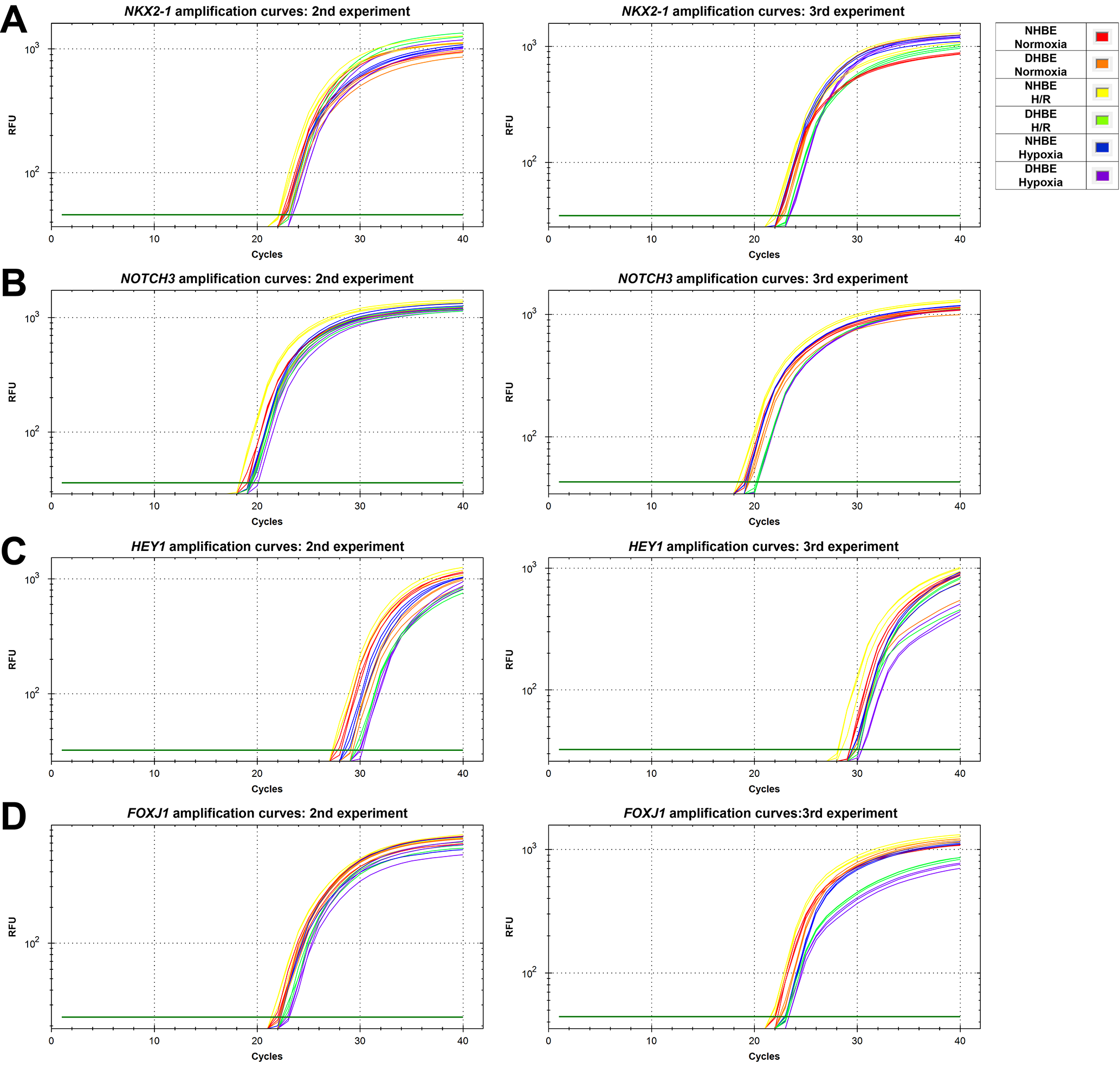

Supplement: FIGURE S3 — Real-time qPCR amplification curves of NKX2-1, NOTCH3, HEY1 and FOXJ1 mRNAs in the ALI-cultured NHBE and DHBE cells for the second and third independent experiments. [file Image_3.TIF]

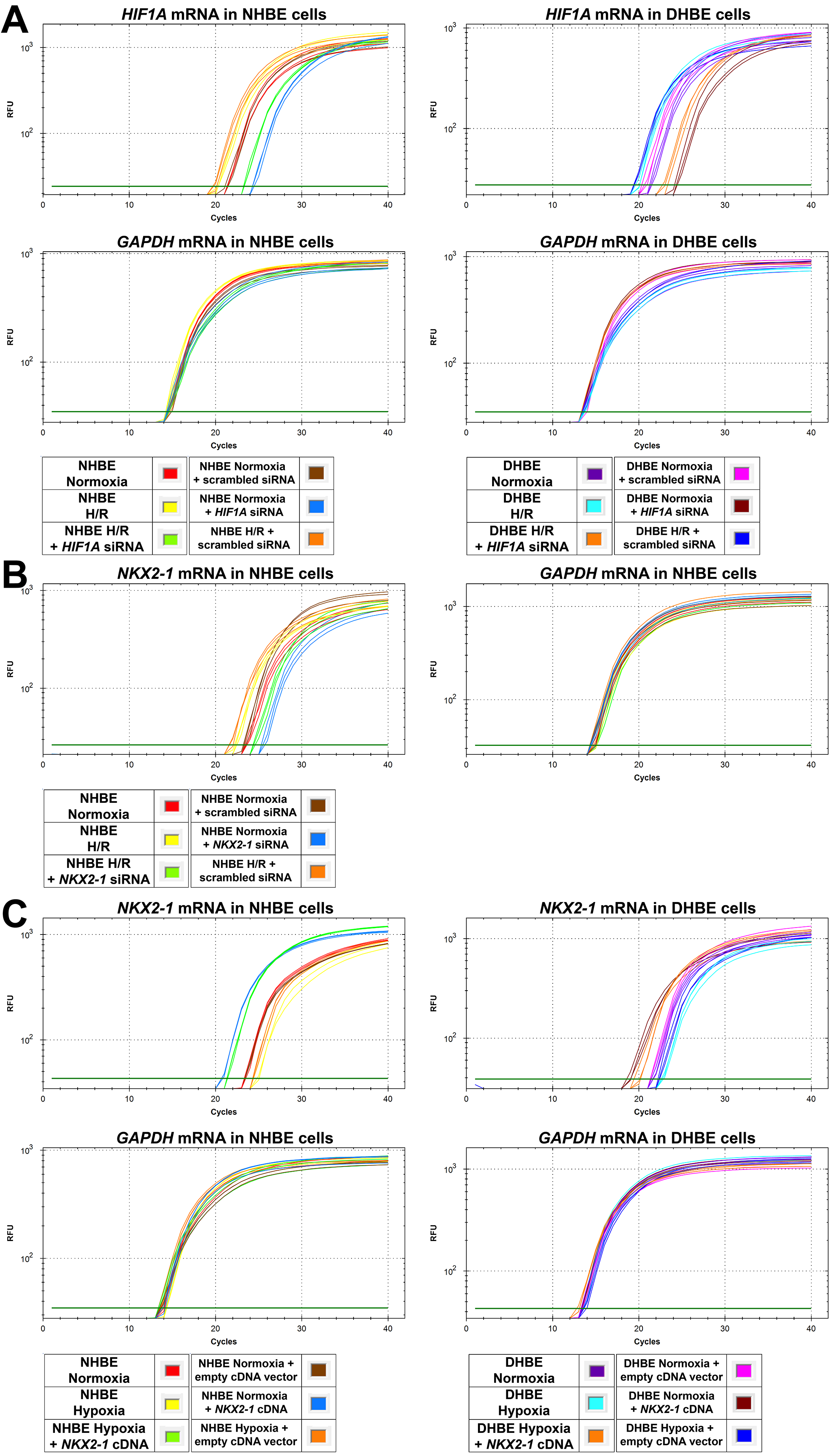

Supplement: FIGURE S4 — Real-time qPCR amplification curves of HIF1A and NKX2-1 mRNAs in the ALI-cultured NHBE and DHBE. (A) Comparison of the HIF1A mRNA levels with scrambled siRNA or HIF1A siRNA transfection. (B) Comparison of the HIF1A mRNA levels with scrambled siRNA or NKX2-1 siRNA transfection. (C) Comparison of the HIF1A mRNA levels with scrambled siRNA or NKX2-1 cDNA transfection. [file Image_4.TIF]

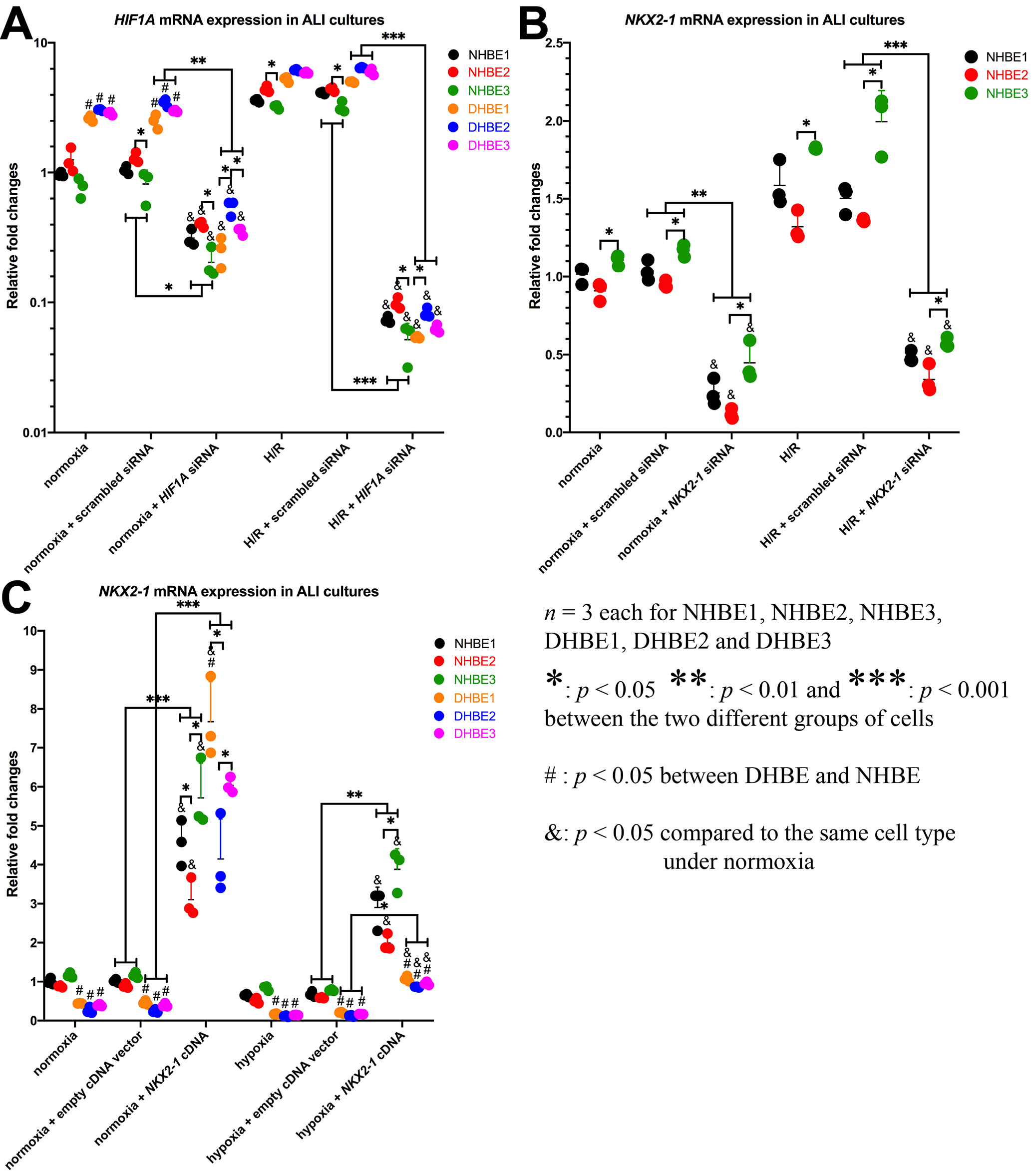

Supplement: FIGURE S5 — Statistical dot plots showing HIF1A and NKX2-1 mRNA levels in the ALI-cultured NHBE and DHBE cells transfected with HIF1A siRNA, NKX2-1 siRNA or NKX2-1 cDNA. The singlet asterisk (∗) indicates p < 0.05 and the doublet asterisk (∗∗) indicates p < 0.01 as compared between the two different groups of cells within the same type (i.e., NHBE2 vs. NHBE3 or DHBE1 vs. DHBE2). The hashtag (#) indicates p < 0.05 when comparing the DHBE tissues with the NHBE tissues cultured under the same oxygen tension, and the ampersand (&) indicates p < 0.05 when compared to the same type of cells cultured under normoxia. [file Image_5.TIF]

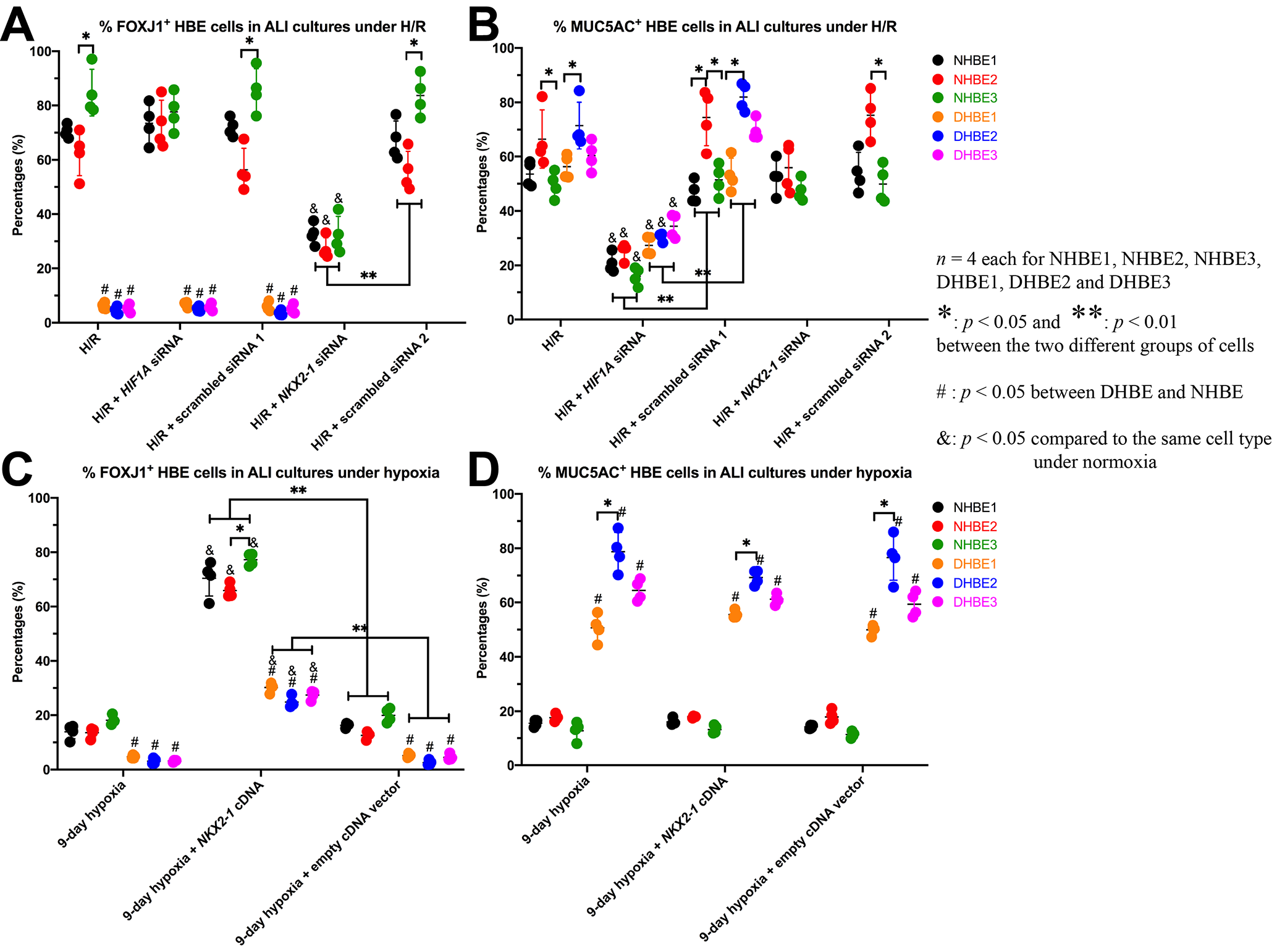

Supplement: FIGURE S6 — Statistical dot plots showing the percentages of FOXJ1 + and MUC5AC + NHBE and DHBE cells in the ALI cultures under intermittent H/R or consecutive hypoxia. The singlet asterisk (∗) indicates p < 0.05 and the doublet asterisk (∗∗) indicates p < 0.01 as compared between the two different groups of cells within the same type (i.e., NHBE2 vs. NHBE3 or DHBE1 vs. DHBE2). The hashtag (#) indicates p < 0.05 when comparing the DHBE tissues with the NHBE tissues cultured under the same oxygen tension, and the ampersand (&) indicates p < 0.05 when compared to the same type of cells cultured under normoxia. [file Image_6.TIF]

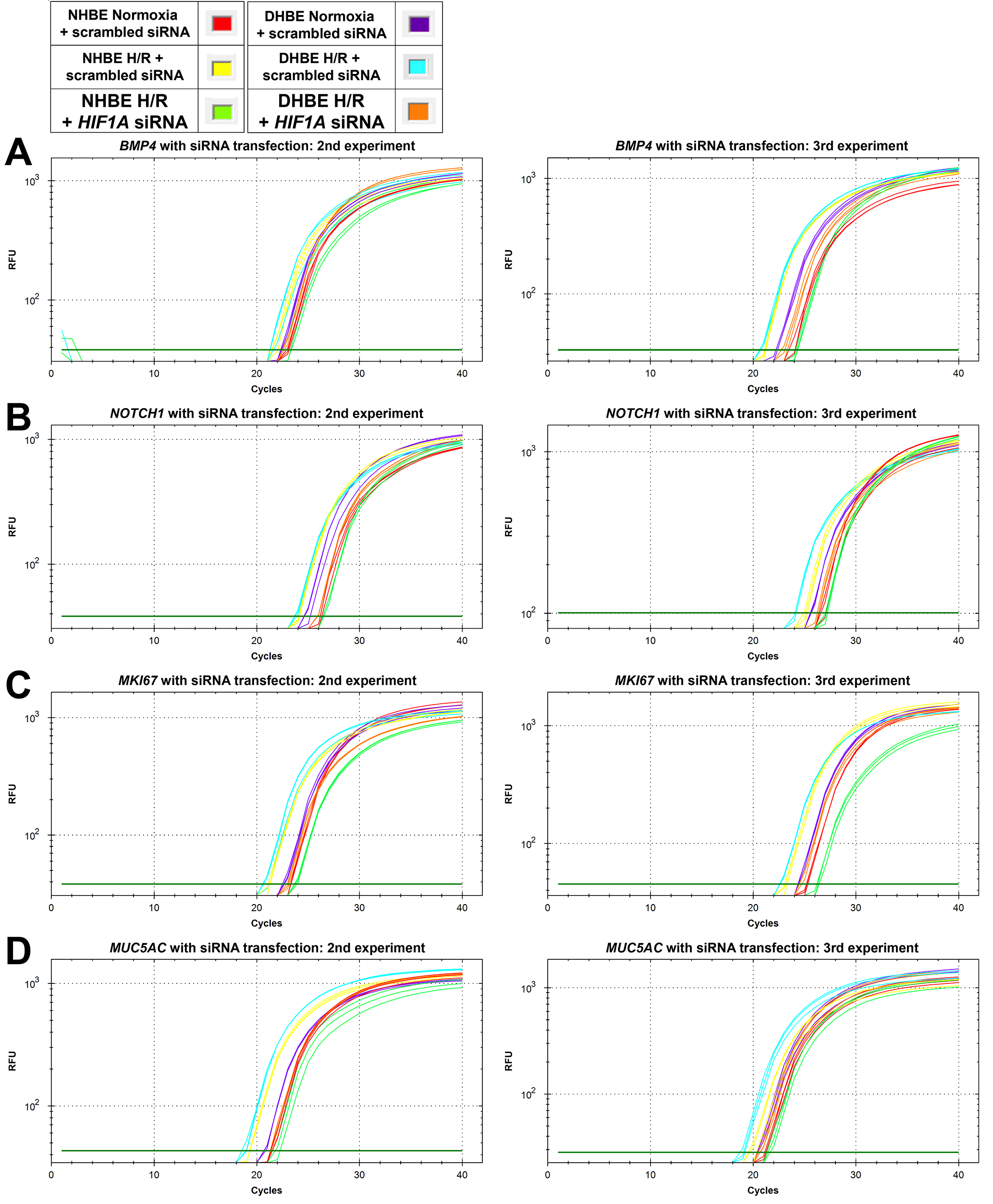

Supplement: FIGURE S7 — Real-time qPCR amplification curves of BMP4, NOTCH1, MKI67, and MUC5AC mRNAs in the ALI-cultured NHBE and DHBE cells transfected with HIF1A or scrambled siRNA for the second and third independent experiments. [file Image_7.TIF]

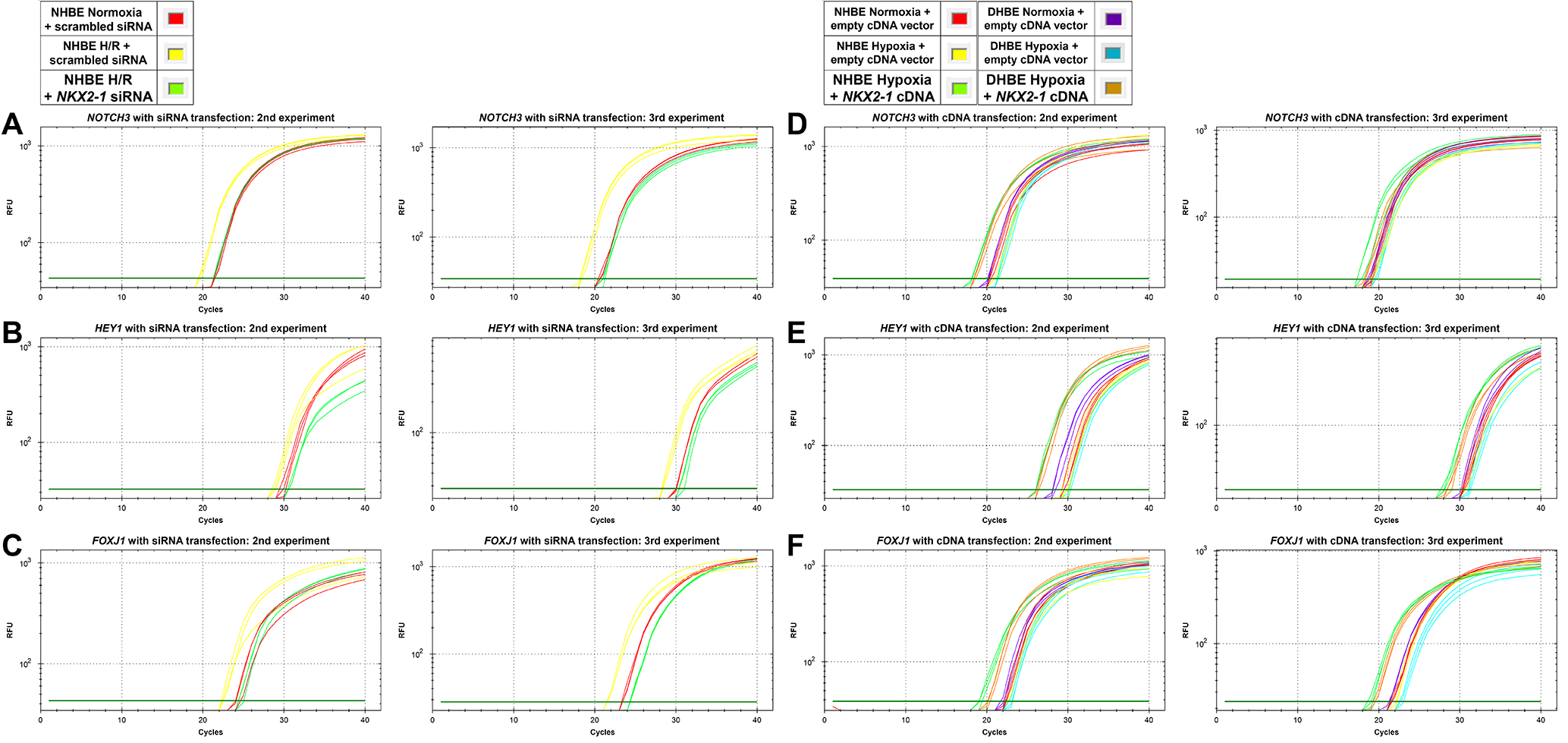

Supplement: FIGURE S8 — Real-time qPCR amplification curves of NOTCH3, HEY1, and FOXJ1 mRNAs in the ALI-cultured NHBE and DHBE cells transfected with NKX2-1 siRNA or NKX2-1 cDNA for the second and third independent experiments. [file Image_8.TIF]

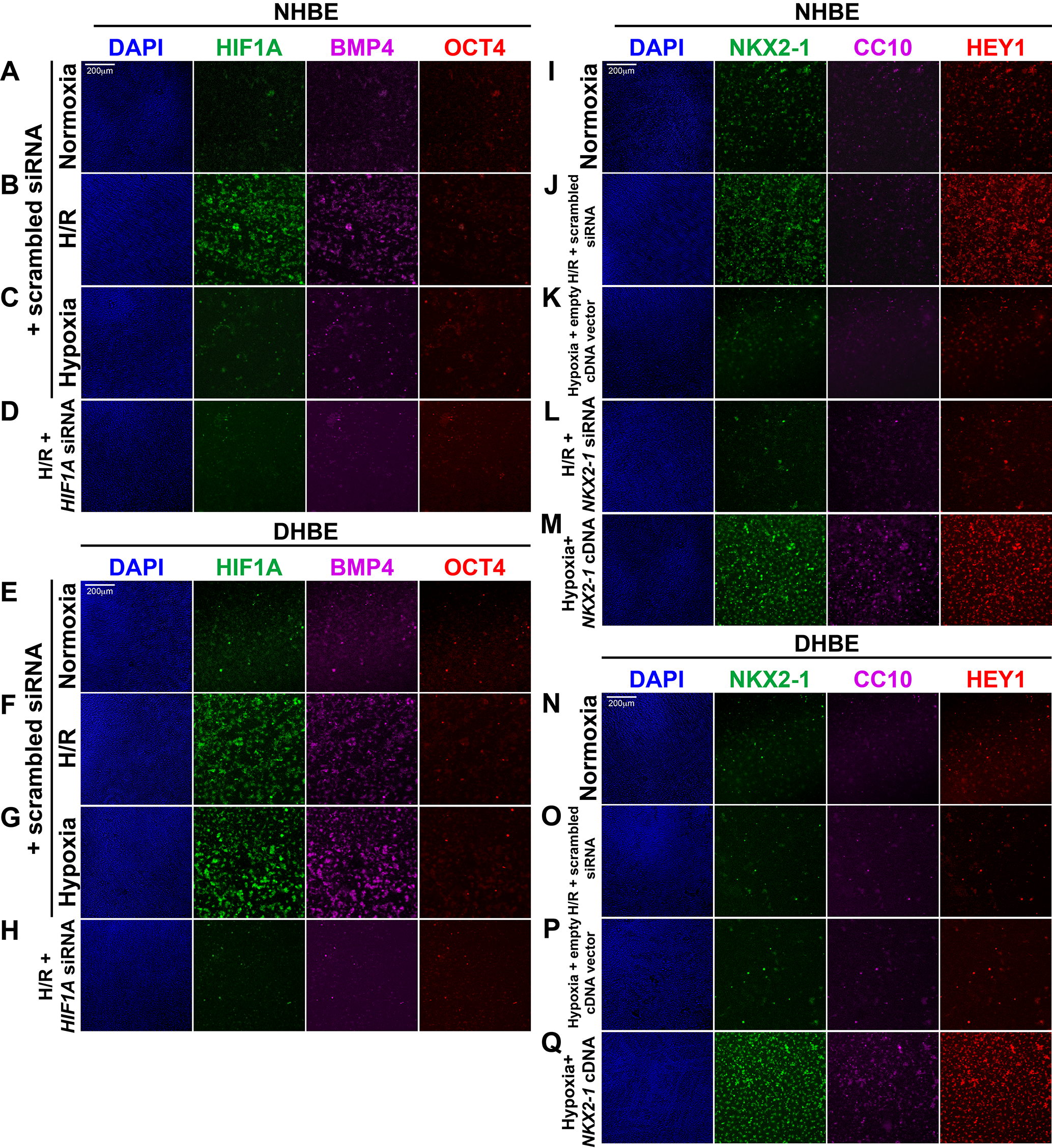

Supplement: FIGURE S9 — Colocalization and concordant regulation of the immunofluorescence signals of HIF1A, BMP4 and OCT4 proteins, and of the immunofluorescence signals of NKX2-1, CC10 and HEY1 proteins in both ALI-cultured NHBE and DHBE cells. (A−H) Triple immunofluorescence staining for HIF1A (green), BMP4 (magenta) and OCT4 (red) in the ALI cultures of differentiated NHBE cells (A−D) and DHBE cells (E−H) revealed colocalization of HIF1A, BMP4 and OCT4 proteins in the same HBE cells. (I−Q) Triple immunofluorescence staining for NKX2-1 (green), CC10 (magenta) and HEY1 (red) in the ALI cultures of differentiated NHBE cells (I−M) and DHBE cells (N−Q) revealed colocalization of NKX2-1, CC10 and HEY1 proteins in the same HBE cells. The scale bars in (A,E,I,N) all represent 200 μm and respectively apply to (A–Q). [file Image_9.TIF]

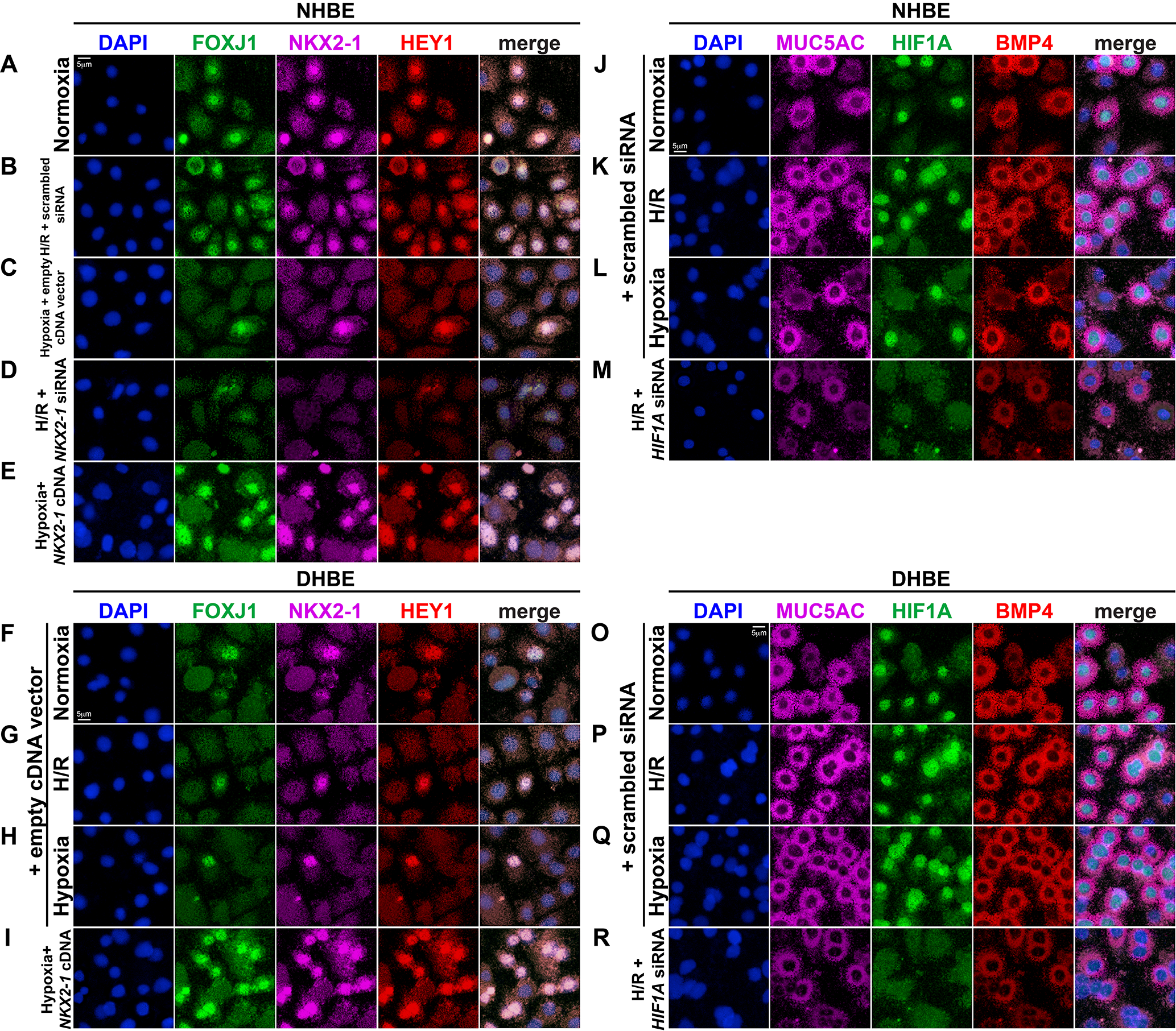

Supplement: FIGURE S10 — Colocalization of the immunofluorescence signals of FOXJ1, NKX2-1 and HEY1 proteins, and of the immunofluorescence signals of MUC5AC, HIF1A and BMP4 proteins in both ALI-cultured NHBE and DHBE cells. (A−I) Triple immunofluorescence staining for FOXJ1 (green), NKX2-1 (magenta) and HEY1 (red) in the ALI cultures of differentiated NHBE cells (A−E) and DHBE cells (F−I) revealed colocalization of FOXJ1, NKX2-1 and HEY1 proteins in the same HBE nuclei. (J−R) Triple immunofluorescence staining for MUC5AC (magenta), HIF1A (green) and BMP4 (red) in the ALI cultures of differentiated NHBE cells (J−M) and DHBE cells (O−R) revealed colocalization of MUC5AC, HIF1A, and BMP4 proteins in the same HBE cells. The scale bars in (A,F,J,O) all represent 5 μm and respectively apply to (A–R). [file Image_10.tif]

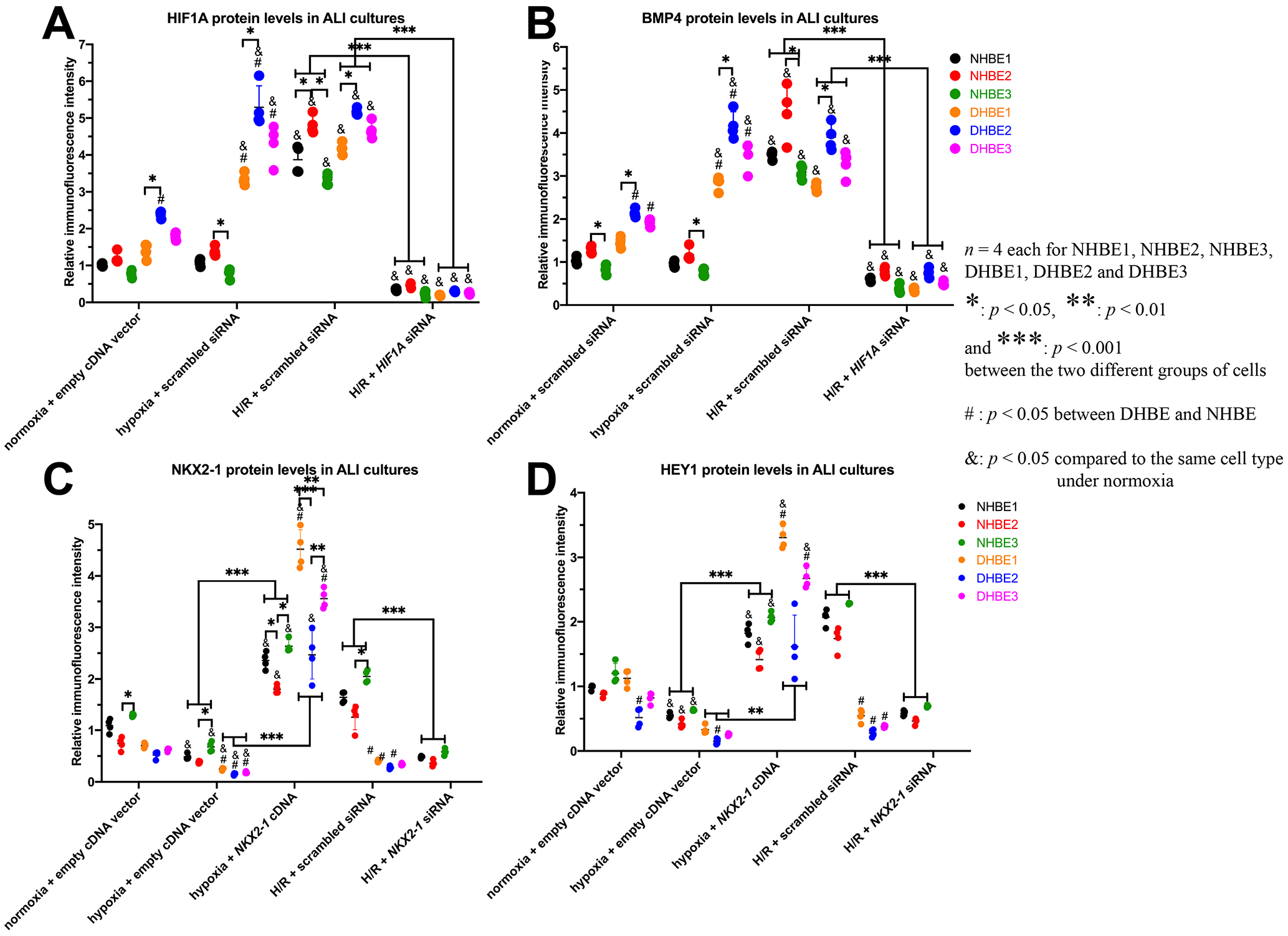

Supplement: FIGURE S11 — Statistical dot plots of the relative protein levels of HIF1A, BMP4, NKX2-1 and HEY1 in different groups of NHBE and DHBE cells in the ALI cultures. Comparison of the relative immunofluorescence intensities of HIF1A (A), BMP4 (B), NKX2-1 (C), and HEY1 (D) in the immunostaining analyses in the ALI cultures between three different groups of NHBE cells (NHBE1, NHBE2 and NHBE3) and three different groups of DHBE cells (DHBE1, DHBE2 and DHBE3). The singlet asterisk (*) indicates p < 0.05, the doublet asterisk (**) indicates p < 0.01, and the triplet asterisk (***) indicates p < 0.001 as compared between the two different groups of cells within the same type (i.e., NHBE2 vs. NHBE3 or DHBE1 vs. DHBE2). The hashtag (#) indicates p < 0.05 when comparing the DHBE tissues with the NHBE tissues cultured under the same oxygen tension, and the ampersand (&) indicates p < 0.05 when compared to the same type of cells cultured under normoxia. [file Image_11.TIF]

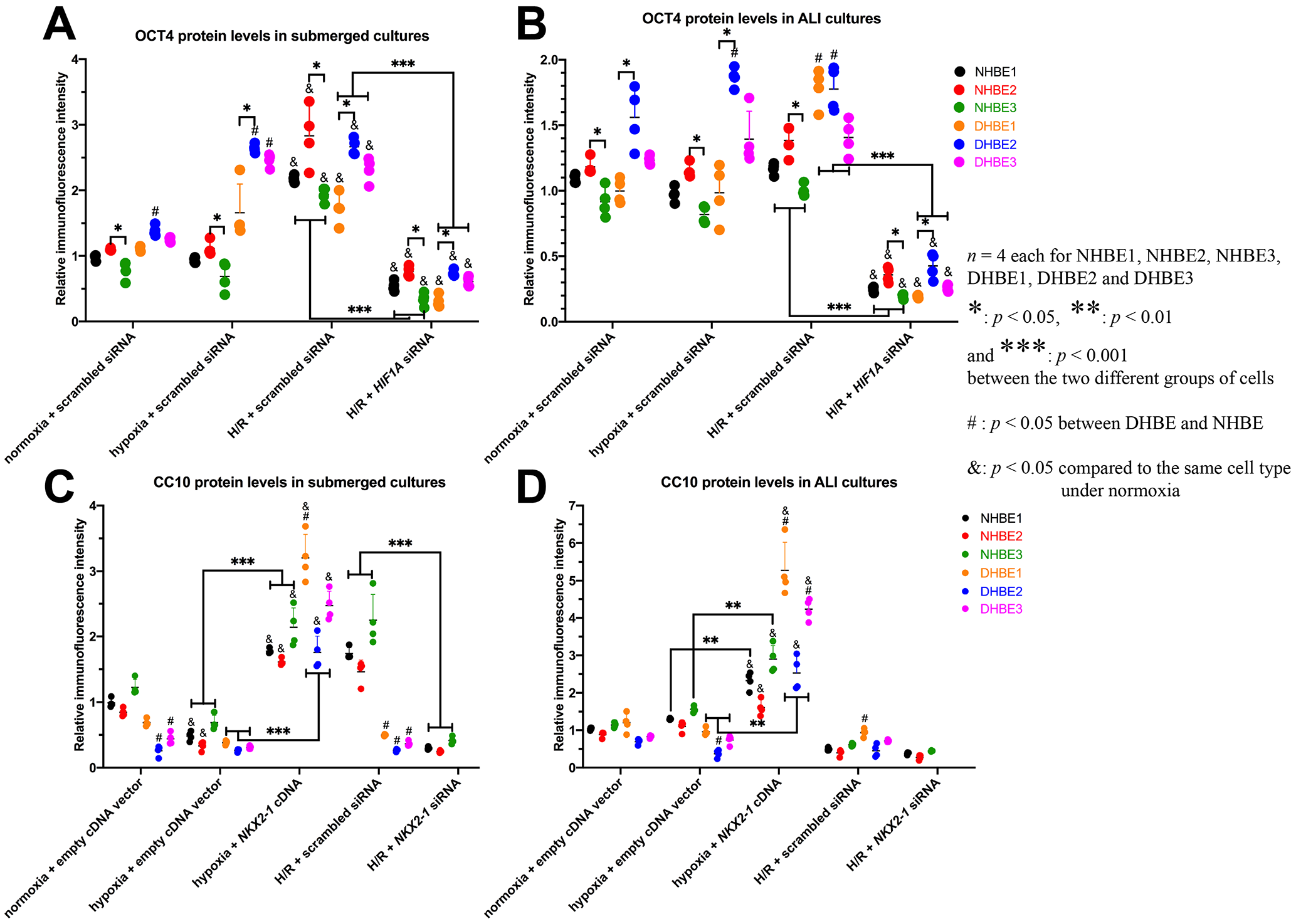

Supplement: FIGURE S12 — Statistical dot plots of the relative protein levels of OCT4 and CC10 in different groups of NHBE and DHBE cells in the ALI and submerged cultures. (A,B) Comparison of the relative immunofluorescence intensities of OCT4 in the submerged (A) and ALI (B) cultures between three different groups of NHBE cells (NHBE1, NHBE2 and NHBE3) and three different groups of DHBE cells (DHBE1, DHBE2 and DHBE3). (C,D) Comparison of the relative immunofluorescence intensities of CC10 in the submerged (C) and ALI (D) cultures between three different groups of NHBE cells (NHBE1, NHBE2 and NHBE3) and three different groups of DHBE cells (DHBE1, DHBE2 and DHBE3). The singlet asterisk (*) indicates p < 0.05, the doublet asterisk (**) indicates p < 0.01, and the triplet asterisk (***) indicates p < 0.001 as compared between the two different groups of cells within the same type (i.e., NHBE2 vs. NHBE3 or DHBE1 vs. DHBE2). The hashtag (#) indicates p < 0.05 when comparing the DHBE tissues with the NHBE tissues cultured under the same oxygen tension, and the ampersand (&) indicates p < 0.05 when compared to the same type of cells cultured under normoxia. [file Image_12.TIF]
